# Technological variability at Sibudu Cave: The end of Howiesons Poort and reduced mobility strategies after 62,000 years ago

**DOI:** 10.1371/journal.pone.0185845

**Published:** 2017-10-05

**Authors:** Paloma de la Peña, Lyn Wadley

**Affiliations:** 1 Evolutionary Studies Institute, University of the Witwatersrand, Johannesburg, South Africa; 2 School of Geography, Archaeology and Environmental Studies, University of the Witwatersrand, Johannesburg, South Africa; Max Planck Institute for the Science of Human History, GERMANY

## Abstract

We evaluate the cultural variation between the youngest Howiesons Poort layer (GR) and the oldest post-Howiesons Poort layers (RB-YA) of Sibudu Cave (KwaZulu-Natal, South Africa). We first conducted a technological analysis, secondly we performed a cladistic study with all the technological traits and, finally, we compare the technological variability with other data from Sibudu (ochre, micromorphology, fauna and plant remains). The synapomorphies of the cladistical analysis show numerous lithic technological changes between the youngest Howiesons Poort and the oldest post-Howiesons Poort layers as previously concluded. However, some technological strategies that are present, yet uncommon, in the Howiesons Poort become abundant in the overlying layers, whereas others that were fundamental to the Howiesons Poort continue, but are poorly represented in the overlying layers. We further show that lithic technological strategies appear and disappear as pulses in the post-Howiesons Poort layers studied. Among the most notable changes in the post-Howiesons Poort layers is the importance of flake production from discoidal knapping methods, the unstandardized retouched pieces and their infrequent representation, and the higher than usual frequency of grindstones. We evaluate various hypotheses to explain the transformation of a Howiesons Poort formal industry to a more ‘expedient’ assemblage. Since no marked environmental changes are contemporary with the technological transformation, a change in residential mobility patterns seems a plausible explanation. This hypothesis is supported by the changes observed in stratigraphy, lithic technology, site management, ochre and firewood collection.

## Introduction

Traditionally in Middle Stone Age studies, the Howiesons Poort has been lionized as a precocious and outstanding industry, with a strong blade based assemblage and backed implements (amongst which the most iconic are segments) [1] attributed to a variety of functions [2–6]. Moreover, together with putatively advanced lithic strategies, this technocomplex includes very distinctive material culture, such as worked bone (at Sibudu), engraved ochre (at Blombos, Klein Kliphuis, Klasies and Sibudu), and ostrich eggshell engravings (at Diepkloof, and Klipdrift) [7–13]. Consequently, some scholars consider the Howiesons Poort as exceptional within the Middle Stone Age sequence [14]. In contrast, the rest of the Middle Stone Age (except Still Bay industries) has attracted less attention (see for a review of the Middle Stone Age [15–17]). This has led to the naming of some industries according to their relationship to the Howiesons Poort, for example, the post-Howiesons Poort. Conard and colleagues [18, 19] criticize the name post-Howiesons Poort because it defines these lithic assemblages by their lack of Howiesons Poort tools rather than by their own idiosyncratic attributes.

In this paper we evaluate the cultural variation between the youngest layer associated with the Howiesons Poort [20] and the oldest post-Howiesons Poort layers of Sibudu Cave. A similar attempt was made in other Middle Stone Age sequences such as Rose Cottage [21], Klein Kliphuis [22] and Diepkloof [23].

In order to evaluate the putative change between these two supposedly different technocomplexes, we conduct a traditional technological study using the *chaîne opératoire* (together with some statistical inputs to control the qualitative results) and evolutionary methodologies. Moreover, we investigate the two Sibudu assemblages from a phylogenetic point of view and we perform a cladistic analysis with technological attributes. Therefore, first we use a descriptive technological (quantitative and qualitative) approach, in order to present all the variants of the lithic technology in the older and younger layers. Secondly, a cladistic analysis is performed. This analysis allows us to describe accurately the degree of variation between the groups of assemblages. Furthermore, the technological information from this cladistics analysis is evaluated together with other archaeological evidence such as the micromorphology (that indirectly informs us about site management and the intensity of occupations), the ochre and the environmental proxies. The combined data enable us to evaluate the putative transition between Howiesons Poort and younger layers at Sibudu.

The layers that we present and compare here include a Howiesons Poort one, the Grey Rocky (GR) layer, and the post-Howiesons Poort overlying layers from Reddish Brown (RB) to Yellow Ash (YA). GR was recently analyzed in a detailed technological study that demonstrated why its assemblage is Howiesons Poort [20]. The overlying layers (upward of RB) have been variously named post-Howiesons Poort [24], ‘Sibudu’ technocomplex’ [25] and ‘Sibudan’ [18, 19]. The assemblage that we study here (from RB to YA) is not the same, technologically, as the younger Sibudan higher in the Sibudu sequence. Therefore, we retain post-Howiesons Poort as an informal label for the occupations immediately following on the hiatus that separates them from the Howiesons Poort.

This is the first time that the final Howiesons Poort layers are compared with the overlying layers in a holistic technological study.

The lithics from GR are briefly summarized here. See de la Peña [20] for a detailed technological description and the methodological protocol (qualitative attribute analysis and basic statistical tests) that will enable future comparison of this Howiesons Poort lithic assemblage with other Middle Stone Age lithic assemblages in Sibudu as well as Howiesons Poort sequences at other sites.

In this paper we go a step further and compare lithics from layer GR with lithics from overlying layers RB to YA to investigate technological differences and similarities. In other words, we attempt to describe accurately the lithic technological changes. Moreover, we draw on other site data, as well as on hunter gatherer literature [26–30] to propose hypotheses that explain the change or stasis that we find.

In a preliminary study of Sibudu lithics from one square metre (B5), Cochrane recorded a remarkable change in lithic raw material management between layer GR and the overlying layers, RB to YA [31, 32]. Therefore, based on these lithic raw material indicators, we selected layers RB to YA for our technological study here.

### Sibudu background

**Fig 1:**
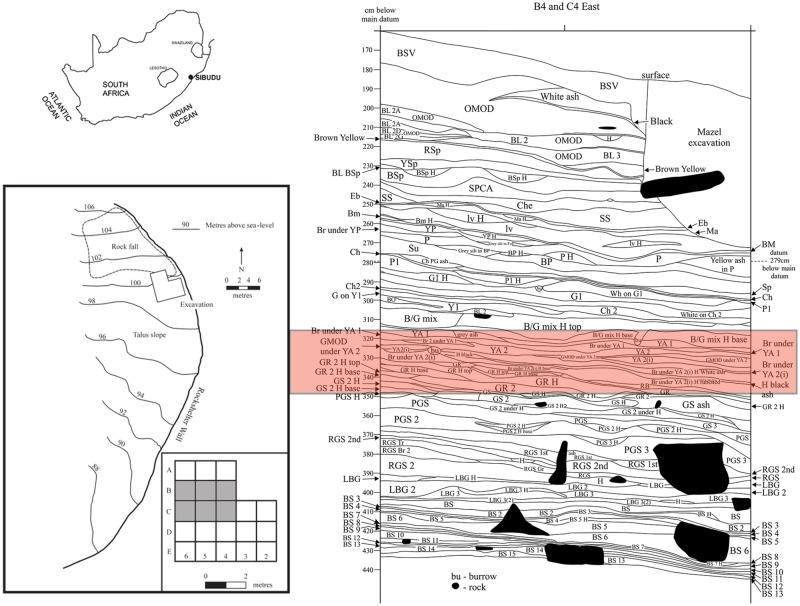
Location of Sibudu. On top left, location of Sibudu (29.522627S, 31.085895E). On the bottom left, Plan of Sibudu. This schematic map was made on the basis of a topographic map of Southern Africa, source: Maps at the CIA (public domain): https://www.cia.gov/library/publications/the-world-factbook/index.html. On the right, stratigraphy of the east face with the layers studied in this paper highlighted in red.

Sibudu is located approximately 40 km north of Durban, and about 15 km inland of the Indian Ocean, on a steep cliff overlooking the uThongathi River (29.522627°S, 31.085895°E)(Fig 1). The shelter is 55 m long and 18 m in breadth and has a long occupation sequence with several layers and features corresponding to the pre-Still Bay, Still Bay, Howiesons Poort, post-Howiesons Poort, late Middle Stone Age, final Middle Stone Age and Iron Age. All the data reported here come from six square metres (squares B4, B5, B6, C4, C5 and C6) of Wadley’s excavations in the deep sounding.

### Stratigraphy

Sibudu’s stratigraphy is predominantly anthropogenic and ash is a major component of sediments throughout the sequence [33]. The layer attributed to the Howiesons Poort that we discuss here is GR which is light, brownish-grey silt. In square B6, layer GR occurs in rock fall. GR2 is an artificial spit to divide the layer. The age of the GR layer is 61.7±2 ka, obtained from single grain optically stimulated luminescence on sediment from GR2 [34]. The layers presented and studied here are attributed elsewhere to the post-Howiesons Poort [24] or Sibudan [18, 19]. They are Reddish Brown (RB), Brown under Yellow Ash 2(i)(BYA2i), Yellow Ash 2(i) (YA2i), Brown under Yellow Ash 2 (BYA2), Mottled Grey under Yellow Ash 2 (Mottled Grey), Yellow Ash 2 (YA2), Grey under Yellow Ash (GYA), Brown under Yellow Ash (BYA) and Yellow Ash (YA) (Fig 1). These layers have not yet been dated and they therefore have an age that lies somewhere between the GR2 age of 61.7 ± 2 ka and the B/Gmix age of 58.2 ± 2.4 ka. The B/Gmix age is part of a suite of six 58 ka ages from a metre-deep stratigraphic column. These Sibudu ages were calculated from single grain optically stimulated luminescence [35] and the technique is not fine-grained enough to measure small age differences. Thus the metre-deep sequence of 58 ka old occupations may represent 1000 years or a few dozen years. The weighted mean of the six ~58 ka ages is 58.5 ± 1.4 ka [35]. All layers considered in this paper are described in detail in S1 File.

In general, Sibudu sediments with ages older than 62 ka ago (incorporating Howiesons Poort lithic assemblages) are shades of grey, and visually the strata appear more homogeneous than those in the younger part of the sequence (Fig 1). Indeed, there is a sharp visual contrast between the older and younger layers. Layers BYA2i to YA are characterized by vivid colouring, mottling and fine lenses that often present at the scale of millimetres. The finely separated layers give the appearance of repeated occupations within a space of time that may be measured in tens or hundreds rather than thousands of years.

The geoarchaeology demonstrates, as is the case with botanical evidence, that there is unusually fine resolution of data at the site. The layering at 58 ka ago is frequently the result of repeated burning of plant bedding material [36, 37]. BYA2i comprises multiple layers of laminated fibrous charcoal and laminated phytoliths [37], typical of burnt bedding layers [36]. The phytolith layers in BYA2i contain secondary phosphates and some plant layers are separated by thin layers of clay [38]. BYA2i appears to represent repeated assembly and burning of bedding, implying intensive site use, repeated occupations and/or long visits at the site. The different sediment formation and preservation in the Howiesons Poort layers suggests that the site was used differently then and that there may have been more time between re-occupations of Sibudu prior to 62 ka ago than was the case 58 ka ago.

Results from FTIR spectroscopy point to less variation in the sediment compositions of GR and the deeper layers than in younger layers above GR [39]. The 47 sediment samples from GR and the underlying sediments comprise about one dozen compositions whereas the younger strata are more complex and can be separated into nearly three dozen compositions [39]. The trampling in GR has caused some of the homogenization, but the moist conditions that seem to have prevailed during the Howiesons Poort may have resulted in greater diagenesis of organic material.

### Palaoenvironmental proxies

*Podocarpus* spp. (Yellowwood) charcoal was recovered most commonly from the GR2 and GR layers together with Proteaceae, Asteraceae, and Rubiaceae/Aponynaceae [40]. The presence of *Podocarpus* spp. amongst the firewood in hearths suggests that, at the time, evergreen forest may have extended from the river to the cliff around Sibudu. The expanse of forest may have been less by 58 ka ago. Many seeds, probably from *Olea* sp. (wild olive), were found in the YA layers, but in the earlier layers these seeds were rare fragments. In contrast, Cyperaceae seeds occur in all Sibudu layers, probably because of the repeated building of bedding from sedges collected at the river near the shelter [41]. No pollen was recovered from layer YA [42] and the YA and YA2 layers also do not have much evidence for laminated burnt bedding.

The evergreen forest in the Howiesons Poort was home to the Gambian giant rat, Geoffroy’s horseshoe bat (*Rhinolophus clivosus*), blue duiker, bush pig and other forest or closed environment animals [43–45]. The giant rat lives only in evergreen forest and woodland where rainfall is greater than 800 mm annually [46]:193 and the bat also requires high humidity [47]:36. Amongst other species found in the closed, especially forested, Howiesons Poort habitats are small ungulates, in particular the blue duiker [44] and these, together with small carnivores, may have fallen prey to snares [48]. The occupations directly after the Howiesons Poort do not reflect an immediate change in faunal pattern. Instead they demonstrate a gradual shift to a preference for large plains game like zebra [40], but the shift does not directly correlate with the technological shifts described here and only fully engages in more recent layers of the 58 ka sequence.

The bird assemblage adds useful data even though the layers overlying the Howiesons Poort do not have many bird bones. The Howiesons Poort contains many bird bones (especially from pigeons) and a range of bird species that have several habitats in common, for example, evergreen forest, rocky areas and water [49]. Immediately after the Howiesons Poort, probably at the MIS4/MIS3 transition, there seem to have been drier conditions. The younger layers (RB to BSp) have few bird bones with little taxonomic diversity, but amongst the identified species there is a higher percentage of grassland and lower percentage of evergreen forest species than in the Howiesons Poort [49]. The scarcity of gypsum, but increased presence of calcite, in Sibudu’s Howiesons Poort layers [40] substantiates the interpretation of higher humidity in the shelter before 62 ka ago because calcite is less soluble than gypsum [50]:113.

### Combustion features

Hearths or small patches of burnt bedding are the most common Howiesons Poort combustion features in layer GR [39]. In contrast, more recent layers have extensive laminated combustion features, probably the result of burning plant bedding for site maintenance. Deep, stacked burnt plant material has built up in the younger layers [37, 38]. Differences in the way that fires were managed and ways in which plant bedding was built and disposed of has affected the sedimentation at the site. Goldberg et al. [36] concluded that Howiesons Poort layers at Sibudu contain isolated or single episodes of burning bedding whereas the 58 ka layers contained multiple episodes or stacks of burning bedding. This suggests different site maintenance strategies for the older and younger occupations that might implicate different settlement densities. The Howiesons Poort layers appear to have more trampling than the younger ones and this is probably a primary reason for the homogeneous grey sediment here, though raking out of combustion features has also been suggested [36]. Thick bedding may have lessened the effects of trampling in the 58,000 year old layers. Compression of sediment and diagenesis of organic materials are also factors to be considered in the Howiesons Poort layers.

### Worked bone

Seven pieces of worked bone were found in the GR and GR2 layers and two from layers YA and YA2 [9,10]. The YA tool is a *pièce esquillée* with a chisel-like edge. The flake scar removals are aligned with the tool’s long axis; the splintering was probably caused by using the bone flake as a wedge to split hard material. The second tool (in YA2) is a pressure flaker. Both *pièces esquillées* and pressure flakers were also present in the Howiesons Poort layers, together with smoothers, points, notched pieces and several other bone tool types [9,10]. Thus, there is some continuity in bone tool manufacture and use between the Howiesons Poort and younger lithic assemblages, although there are fewer bone tools and tool types in the latter.

### Ochre

The term ochre is a general one that includes haematite as well as earthy red, yellow, orange and purplish-red rocks. Ochre was sometimes used in Howiesons Poort adhesive recipes for hafting backed tools to shafts [51–54] and also in younger assemblages for attaching retouched points to shafts, presumably as hunting weapons [54,55]. Layers BYA2i to YA have few retouched tools and the use of ochre loaded adhesive has not been observed here because flakes and blades have not yet been examined for residues.

There are two main textures of ochre at Sibudu, clayey and silty, and both occur throughout the sequence. However, the Howiesons Poort layers have higher percentages of soft, clayey ochre pieces than layers BYA2i to YA. These younger layers contain more silty pieces, especially utilised ones. Clayey ochre is best for rubbing directly on skin, hide or other soft products, whereas silty ochre is better when ground to produce ochre powder. However, neither type need necessarily be restricted to one or other task. Silty ochre occurs in a shale outcrop about one kilometre from Sibudu, whereas the origin of the clayey ochre is unknown and it presumably comes from farther afield than the silty ochre. There is thus an observable difference in the way that ochre was collected in the Howiesons Poort compared with later assemblages and it seems likely that ochre collections were made close to the site at 58 ka ago.

### The availability of rocks around Sibudu

The main rock types and minerals knapped in layers GR to YA at Sibudu are: dolerite, hornfels, sandstone, quartz and quartzite. Cryptocrystalline material provides a small component and there are other rock types which appear from RB upwards, such as andesite. The uThongathi River below Sibudu is a source of weathered and river-rolled dolerite and quartzite as well as of small quartz pebbles. The rounded cortex on a number of Sibudu cores and flakes implies a waterborne origin for some dolerite brought to the site, but cores and flakes made from tabular dolerite pieces are also present. Abundant igneous dolerite near Sibudu derives from intrusive Jurassic volcanism, mostly as sills, although a true dolerite dyke lies close to the rock shelter [56] and this seems likely to have been the source of much of the dolerite at the site. Dolerite sills in the area include fine-grained ones like the Mhlasini sill and coarse-grained ones like the Verulam sill [56].

Where dolerite intrudes into shale, there are bands of metamorphic hornfels. Differing temperatures occur in the zone of thermal metamorphism where a dolerite intrusion occurs. Consequently, there are different grades of both hornfels and dolerite. Hornfels is difficult to find in the Sibudu area, but one source is near Verulam, about 15 km from Sibudu.

Sandstone is the ‘local’ rock type at the site, as the Sibudu rock shelter is situated in a sandstone cliff within the Mariannhill Formation of the Natal Group [56]. The sandstone knapped at the site looks identical to the one that is forming the rock shelter. It is quite a coarse grained subtype.

Crystalline quartz appears as river pebbles and as pebbles inside conglomerates in the Verulam area. Some big quartz pieces could come from river pebbles, but this does not seem the case for some other big pieces. Probably quartz dykes were used as a source for this raw material in the past, judging by the macroscopic characteristics of some cortex areas of these pieces. Within the crystalline quartz assemblage two main categories can be distinguished: vein quartz (milky or xenomorph) and crystal quartz (also called hyaline or automorph quartz) [57].

Quartzite appears as river-rolled small pebbles and it is usually medium/ fine grained.

## Methodology

The technological analysis of the stone tool attributes follows the methodology described in de la Peña [20], which was a mix between qualitative and quantitative parameters to control the *chaîne opératoire* results. In addition, we have created a simple classification for the grindstone pieces that are relatively common here. We distinguish round grindstones from oval ones (lozenges), and more rectangular shapes that have ground facets or a single flat surface that is ground. One distinct type is a slab of sandstone that is heavily ground to a straight ridge on one end.

All lithic artefacts (including chips) were examined in order to identify and analyze any features of potential technological importance. The lithic assemblage was divided into four broad analytical categories: (1) cores, (2) blanks without retouch (including core related by-products), (3) retouched blanks and (4) chips. The chips include pieces with a wide range of morphologies that are smaller than 10mm for quartz, since previous studies of the GS layer showed that this cut-off was the most appropriate for this material [20], and smaller than 20mm for the other raw material types. The sample studied from GR was the following: all cores (n = 120), core related by-products (n = 63) and retouched pieces (n = 244) from Wadley’s excavations. In order to understand lithic production better we also analyzed all complete flakes (n = 1091) (see S1 File in [20]). For the RB to YA layers we analyzed: all cores (n = 92), core related by-products (n = 22), retouched pieces (n = 45), complete flakes (n = 806) (see S2 Table) and bipolar blanks (n = 174).

For the phylogenetic analysis we performed a cladistics analysis with the software Mesquite (to create the cladistics datamatrix) [58] and TNT (to produce the cladograms) [59].

A cladogram groups taxa (in our case layers with technological characteristics) depending on the presence of common evolved characters. Thus, the synapomorphies reflected in the nodes of the groups depict the shared technological variations of these layers, whereas the autapomorphies point out the distinctive characters of each layer.

In order to perform the cladistics analysis we used 63 technological and rock type related characters (see S1 Table). We used different technological attributes that we considered relevant related to raw material, knapping methods, frequency of tools, etc. Therefore, before the cladistics analysis we had to analyze the lithic technology to select the main attributes considered in the data-matrix.

To root the trees in the cladistics analysis we used the technological characteristics of another Howiesons Poort layer: Grey Sand (GS) (the main characteristics of this layer were published in [60]).

The cladistics analysis and the trees were conceived of, and used in this study, as a mere tool for describing and assessing variability. In this case technological variability is meant, in the sense applied in many other geographical contexts and archaeological examples (see for example [61, 62]).

The advantage with cladistics is the analysis of all the characters together to propose the hypothesis of relationships. The cladistics analysis is considering presence/absence of technological traits and it can also consider changes in frequencies. Through this analysis the most parsimonious solution of the characters is presented. In this regard, the cladistics is giving an interpretation of how these layers relate between them.

The excavations at Sibudu were undertaken with heritage permit (number 007/09) issued by the South African heritage agency responsible for KwaZulu-Natal, Amafa KwaZulu-Natali. The Sibudu lithics are stored in the Archaeology section of the Evolutionary Studies Institute, second floor, Palaeosciences Building, University of the Witwatersrand. The site code for Sibudu collections is 2931CA 15 and the specific collections described here are curated with 2931CA 15, followed by the layer name (for example, BYA2i, YA2 and YA), the squares excavated and the box contents (in this case: Lithics). The collections are available for study subject to the conditions laid down by the University and the Evolutionary Studies Institute.

## Results of the technological analysis and cladistics analysis combined regarding lithics

After performing the lithic analysis we selected relevant qualitative technological traits and with them we performed a cladistics analysis using data from GS and GR (both attributed to Howiesons Poort in previous studies [20, 57, 62]) and RB to YA, attributed initially to the post-Howiesons Poort [31,32] and, more recently, to the Sibudan [18,19]. See S1 Table of the supplementary material to see the list of attributes that include raw material strategies, main knapping methods, core representation and retouched pieces.

**Fig 2:**
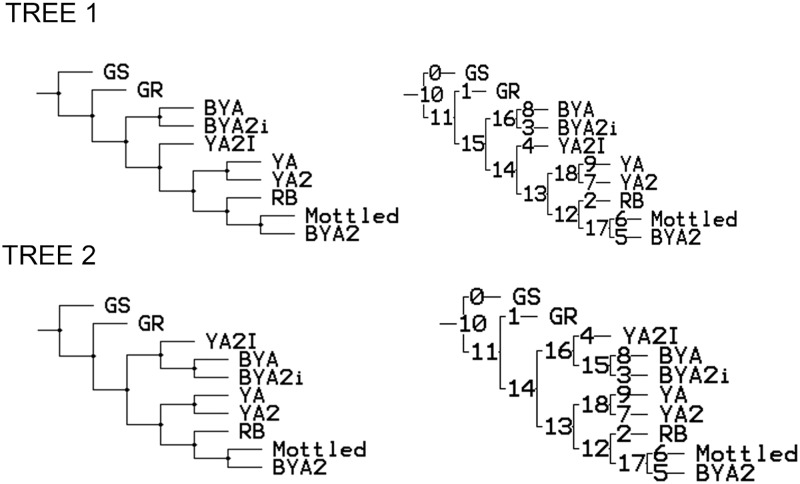
Cladistic trees. On the left, the cladograms with only the layers shown. On the right, the cladograms are represented with the nodes and number of synapomorphies.

After the cladistics analysis two trees were obtained (Fig 2). Both of the trees clearly separate GR from the other eight overlying layers through different synapomorphies. The main difference between the two trees is the position of YA2i. The main reason for its ambiguous position is that few characteristics are available to characterize the layer’s assemblage. For the rest of the layers both trees are essentially identical.

As commented previously, there is a clear difference between GR and the eight uppermost layers. Moreover, there are two big groups within those eight layers: group 1 (BYA and BYA2i) and group 2 (YA, YA2, RB, Mottled and BYA2). The following pairs: BYA-BYA2i, YA-YA2, Mottled-BYA2 form monophyletic groups. As can be seen by comparing the results with the stratigraphy, these monophyletic groups do not show stratigraphic continuity. Although the layers overlying GR show variability, some of them can be associated because the technological cladistics analysis groups the brown sand layers and the yellow ash layers in separate monophyletic pairs. This could mean that there is a correlation between the type of site use and technology, and this is an issue worth exploring in the future.

First, we describe the main technological characteristics of all the layers. The main characteristics derive from the *chaîne opératoire* analysis and the autapomorphies of the cladistics analysis (see S2 File).

After, we describe Tree 1 and its nodes/synapomorphies, because the two cladograms are practically identical.

### Main technological characteristics of each layer using the *chaîne opératoire* analysis and the autapomorphies

In this section we describe the main lithic technological characteristics (using the *chaîne opératoire* analysis) that distinguish each layer. Moreover, for each one of them we highlight the autapomorphies of the cladogram (see the complete list of autapomorphies in S2 File), because these point to distinctive characters.

#### GR

**Fig 3:**
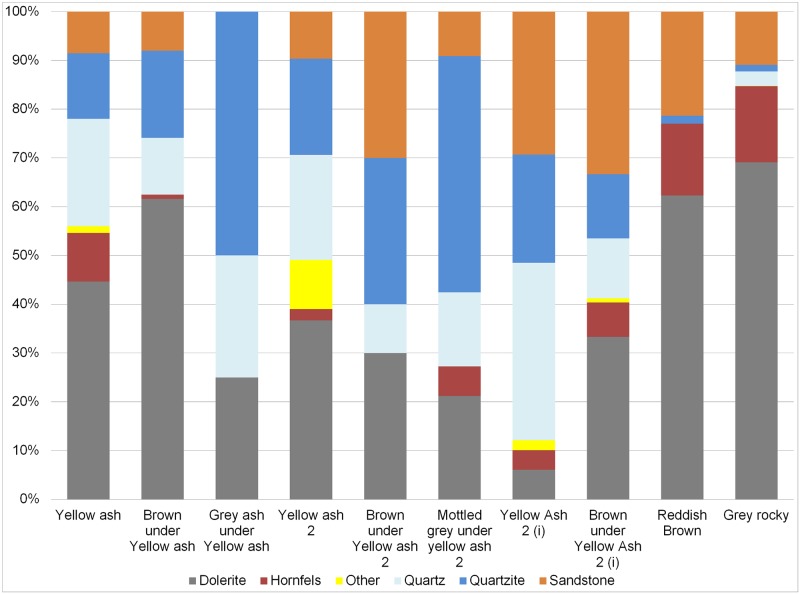
Percentage of rock types for completed flakes >2cm from GR to YA layers.

The main rock types are dolerite, hornfels and sandstone (Fig 3). The principal technological characteristics of this layer were extensively described in [20], but we summarize them here to facilitate their comparison with the upper layers because that is one of the main goals of this paper. A great variety of dolerite and hornfels blade/bladelet and flaking methods occur. Notably present is prismatic blade production, Howiesons Poort core blade/bladelet production, core on flakes and discoidal cores. Moreover, some dolerite discoidal and *Levallois* flakes (both autapomorphies) occur among the non-retouched blanks. The knapping of quartz is also important in this layer, as quartz is the most retouched rock type. Regarding quartz there is discoidal production (autapomorphy), as well as bladelet prismatic production (autapomorphy), and it has been demonstrate that probably some of these cores were recycled as bipolar cores [63]. Also worthy of comment is the presence of flake and blade production in sandstone and of quartz bipolar reduction in quartzite. Finally, this layer is characterized by a large frequency of retouched pieces in comparison with the uppermost layers. Moreover, there is morphotype standardization among backed pieces, which are the most common morphotypes for hornfels and dolerite. In quartz the most represented tools are bifacial pieces [20, 64] (Tables 1 and 2).

**Table 1:** Main technological categories for all layers (from GR to YA).

Layers	Freehand Cores	Bipolar cores	Complete flakes	Bipolar blanks	Retouched pieces	Core related by-products	Total	Litres sediment	Lithics per litre
YA	1	0	141	0	4	5	151	265.25	0.569
BYA	3	2	112	2	4	1	124	136	0,9
YA2	4	16	218	49	8	3	298	330.5	0.9
GMODYA2	1	8	33	37	2	0	81	26.35	3.07
BYA2	1	4	124	6	2	0	137	44.8	3.06
YA2i	3	2	99	5	2	2	113	98	1.15
BYA2i	2	68	124	70	13	4	281	157.25	1.786
RB	2	11	61	6	10	11	101	57.6	1.75
GR	120	49	1091		245	63	1568	503.8	3,1

**Table 2:** Retouched pieces in RB-YA layers.

	YA	BYA	YA2	BYA2	GMODYA2	YA2i	BYA2i	RB	GR
Backed piece	1		1		1		2	1	
Bifacial		2				1	6		
Ind. Retouched piece			1		1		1	1	
Notch	1		3			1			
Resharpening flake			1				2		
Retouched blade		1	1	1				1	
Retouched flake	2			1			2	6	
Strangulated piece								1	
Total	4	3	7	2	2	2	13	10	245
Litres of sediment	265.25	136	330.5	44.8	26.35	98	157.25	57.6	503.8
Volume density	0.02	0.02	0.02	0.05	0.08	0.02	0.08	0.2	0.5

Two grindstones are present in GR, one small lozenge and one large round grindstone (Table 3).

**Table 3:** Grindstones from GR to YA.

Grindstone type	YA	BYA	YA2	BYA2	GMODYA2	YA2i	BYA2i	RB	GR	TOTAL
faceted pebble	1	2		1	1					5
lozenge	1					1			1	3
round		2	3		1		1		1	8
flat grinding surface		1								1
flat and round					1					1
straight ridge		1	1							2
TOTAL	2	6	4	1	3	1	1		2	20

#### RB

**Fig 4:**
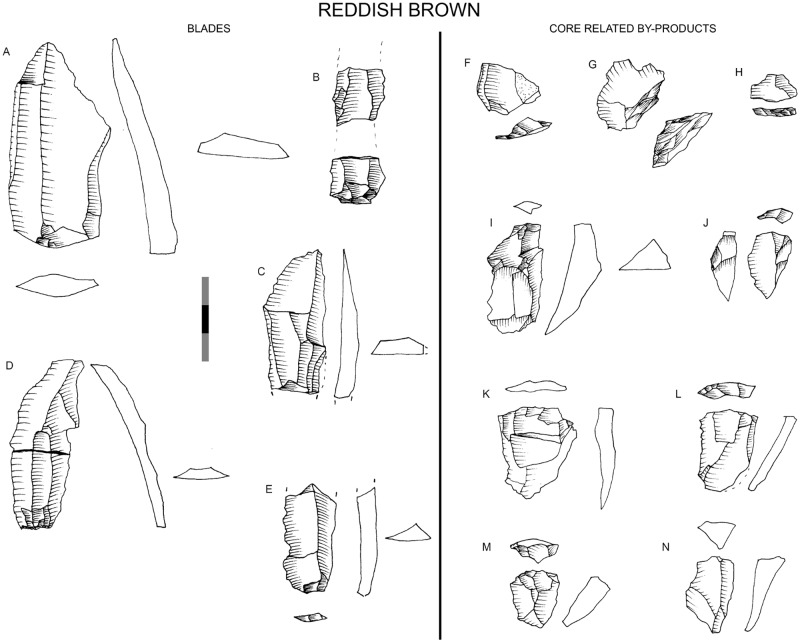
Reddish Brown (RB) technological characteristics. On the left, blade prismatic blanks in dolerite. On the right, core-related by products also in dolerite. Scale 3 cm.

**Fig 5:**
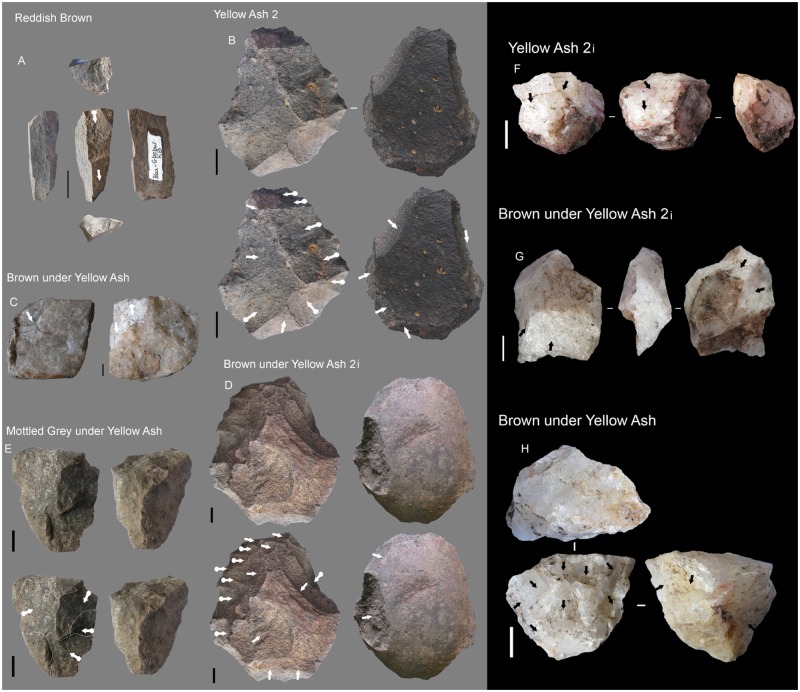
Different types of cores from RB to YA layers. A. Burin-like core in RB. B (dolerite), D (dolerite) and E (quartzite) discoidal cores in YA2, BYA2 and Mottled Grey respectively. C and G. Tested quartzite and quartz cores from BYA and BYA2i respectively. F. Prismatic bladelet quartz core from YA2i. G. Multifacial quartz core from BYA. All scales 1 cm.

The percentage of rock types is similar to GR regarding hornfels (autapomorphy), dolerite and quartzite percentages, although it is remarkable that quartz practically disappears and sandstone gains some importance (Fig 3). Blade prismatic production in dolerite and hornfels continues, mainly represented by non-retouched blanks (Fig 4). In hornfels there is bladelet production and a burin-like core has been recorded (Fig 5, which is also an autapomorphy). It is notable that in this small layer elongated flake production is well represented by blanks and a big end-scraper core. Bipolar production in quartz completely disappears (as it does in all the uppermost layers), whereas quartzite bipolar knapping gains importance (Table 1). The number of blanks and retouched pieces per litre drops notably from 3.1 to 1.75 and from 0.5 to 0.2 (Tables 1 and 2). There is only one piece in dolerite which could be classified as the recycling of a tablet into a segment. This is the first layer where the standardization of morphotypes is completely absent.

#### BYA2i

**Fig 6:**
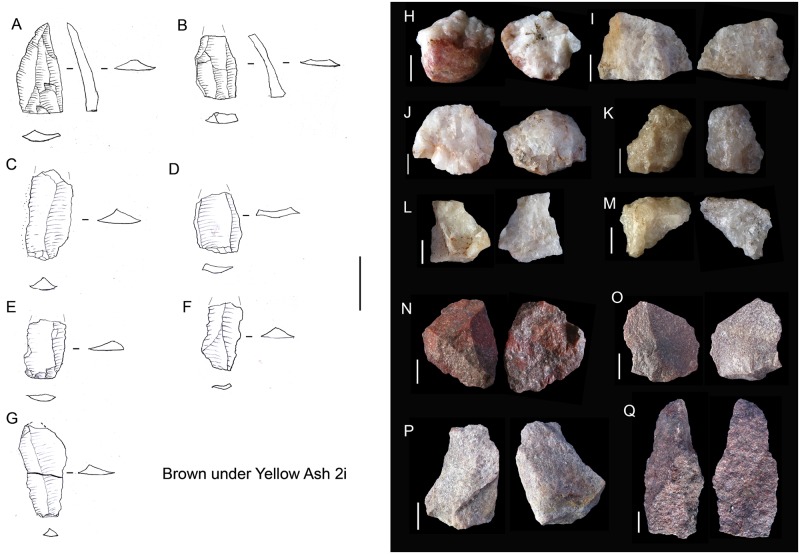
BYA2i non-retouched blanks. On the left, dolerite appointed blades (A and B) and prismatic blades (C-G). Scale 3cm. On the right, discoidal flakes in quartz (H-M), quartzite (N-P) and an elongated flake in sandstone (Q). All scales 1 cm.

**Fig 7:**
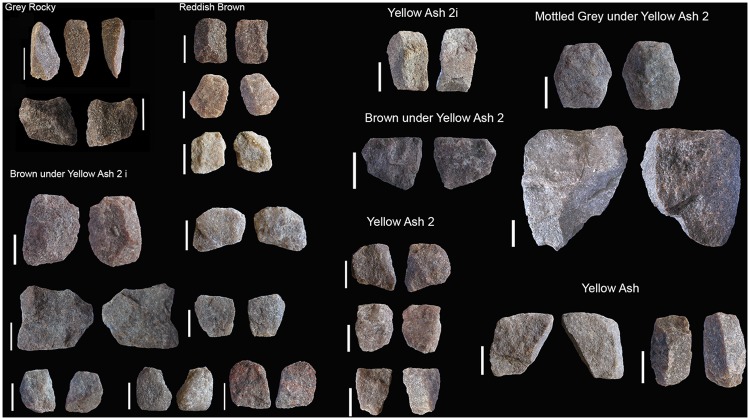
Bipolar cores from GR, RB, BYA2i, YA2i, BYA2, YA2, Mottled Grey and YA. All scales 1 cm.

**Fig 8:**
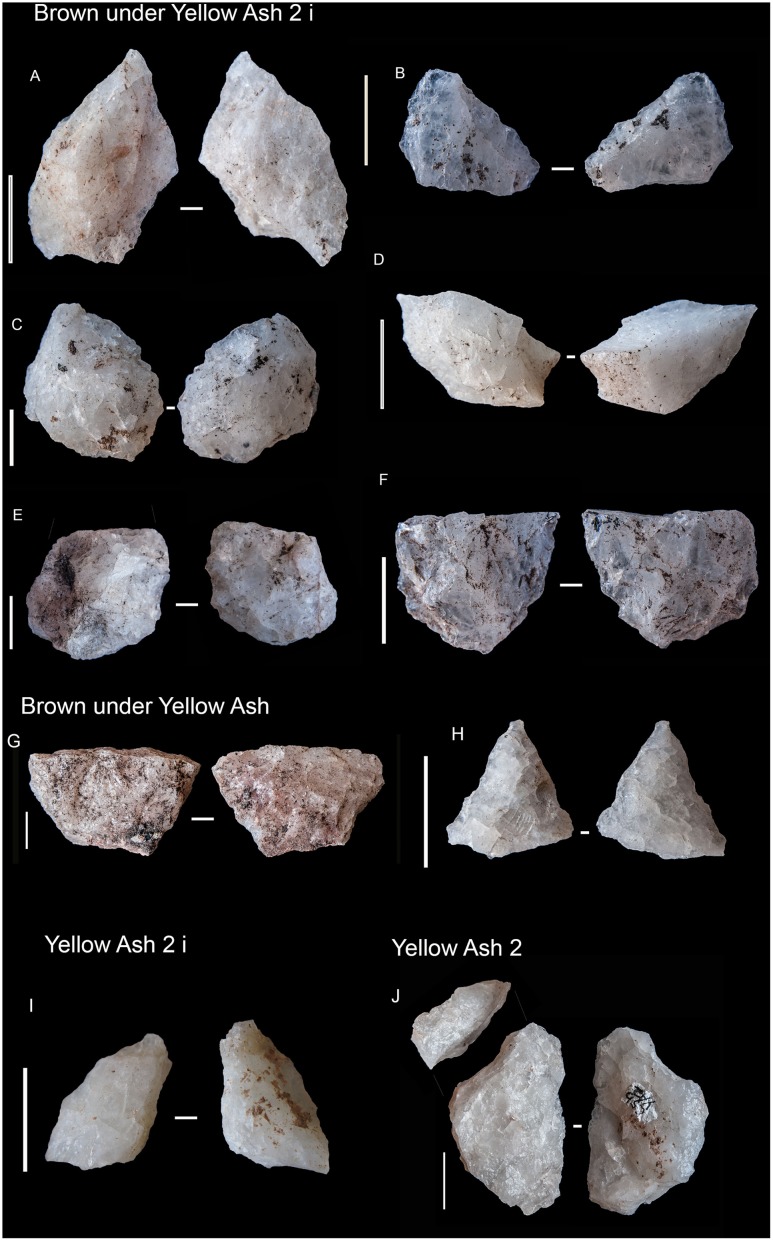
Some of the quartz retouched pieces from RB to YA. A–I. These pieces have been interpreted as bifacial fragments (see also Table 4). J. Quartz notch. All scales 1 cm.

**Fig 9:**
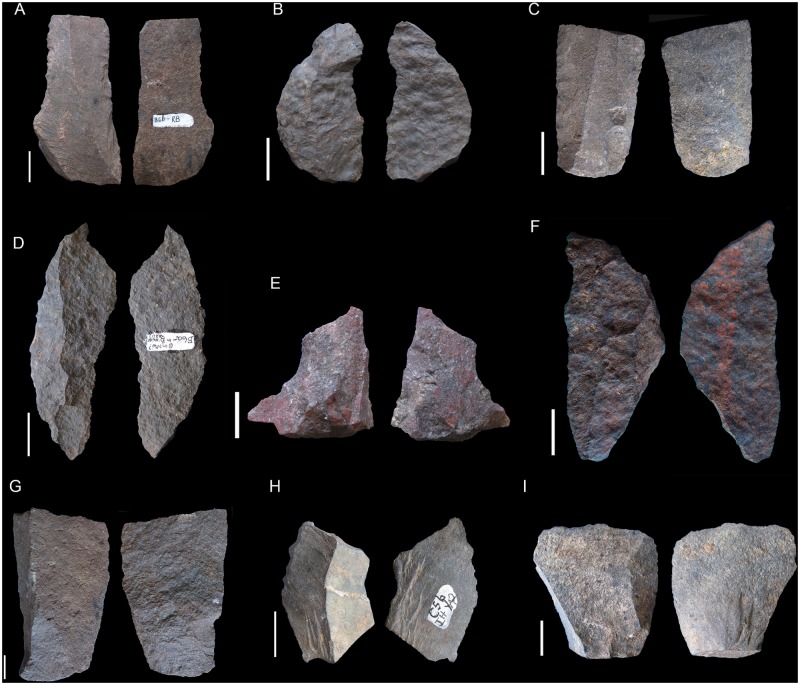
Retouched pieces from RB to YA. A. Retouched hornfels blade from RB. B. Dolerite segment from RB. C. Retouched hornfels blade from BYA. D. Dolerite segment from BYA2i. E. Quartzite notch from YA2i. F. Dolerite segment from Mottled Grey. G. Quartzite notch blade from BYA2. H. Truncated hornfels blade from YA2. I. Retouched hornfels flake from YA.

In this layer the percentage of dolerite begins to drop although, together with sandstone, it is still the main rock type. Moreover, quartz re-appears and gains noticeable importance together with quartzite (Fig 3). It is interesting to note that new rock types appear, such as andesite. In regard to blade production, there is minimal representation of dolerite and hornfels blade prismatic production, which is characterized by some blanks (Fig 6, on the left) and one false semicrest in dolerite (see de la Peña [20] for this terminology). It is also notable that in this layer substantial quartz blade production appears whereas it was not present in the older layers (either RB, GR or GS). It is accompanied by bladelet production (represented by a core, an autapomorphy). There is freehand flake production on quartz, quartzite and sandstone (Fig 6 on the right). The main hallmark of this layer is the importance of quartzite bipolar cores and bipolar blanks (Fig 7), which increase considerably in comparison to the older layers (Table 1), although in those layers quartzite bipolar knapping was present(GR and RB). Regarding the morphotypes, as observed by Will and Conard [19], there is bifacial production (autapomorphy) in and after the Howiesons Poort. In this study we found six new quartz bifacial pieces in BYA2i and other layers (see Tables 2 and 4 and Fig 8A–8F); in all cases they represent a similar morphology to the ones documented in GR and GS [64]. Among the retouched pieces there are also pieces with backed retouch and one of them seems to be a segment (Fig 9D). One round grindstone is present (Table 3).

**Table 4:** Bifacial pieces from layers BYA2i, YA2i and BYA. The x denotes that the piece was not completed.

Layer	Feature	Square	Rock type	Type of blank	Length	Breadth	Thickness
BYA	BYA	B4d	Quartz	Chunk	44.5	27.41	12.93
BYA	BYA	B4b	Quartz	x	x	x	4.07
YA2i	YA2i	C4d	Quartz	x	x	x	x
BYA2i	H1BYA2i	C4b	Quartz	x	x	23.33	11.72
BYA2i	H1BYA2i	C4b	Quartz	Flake	x	23.89	11.5
BYA2i	H1BYA2i	C4a	Quartz	x	x	18.5	6.77
BYA2i	ADBYA2i	C4a	Quartz	x	x	17.49	6.12
BYA2i	H2BYA2i	C4d	Quartz	x	x	14.81	4.22
BYA2i	H1BYA2i	C4a	Quartz	Flake	x	19.04	6.46

#### YA2i

Quartz becomes the most abundant rock type (autapomorphy) surpassing dolerite (autapomorphy), sandstone and quartzite (Fig 3). Dolerite and hornfels decrease notably. As in BYA2i rock types such as andesite appear in addition to quartz, dolerite, hornfels, sandstone and quartzite. Blade prismatic production in dolerite and hornfels is present (autapomorphy), but it is still a very minor component of the non-retouched blanks. The quartz blade production is also represented among the blanks. Regarding the flake production, discoidal quartz production in the form of cores and flakes (autapomorphy), is well-represented. Moreover, sandstone, quartzite and dolerite flake production is also noteworthy. Bipolar knapping in quartzite is only represented by two small cores, and this is in contrast to the high frequencies represented in BYA2i. Among the retouched pieces there is a possible bifacial piece in quartz, (Fig 8I), one big notch in quartzite and a backed piece (autapomorphy); and the most retouched rock type is quartzite (autapomorphy). One small lozenge-shaped grindstone was recovered.

#### BYA2

**Fig 10:**
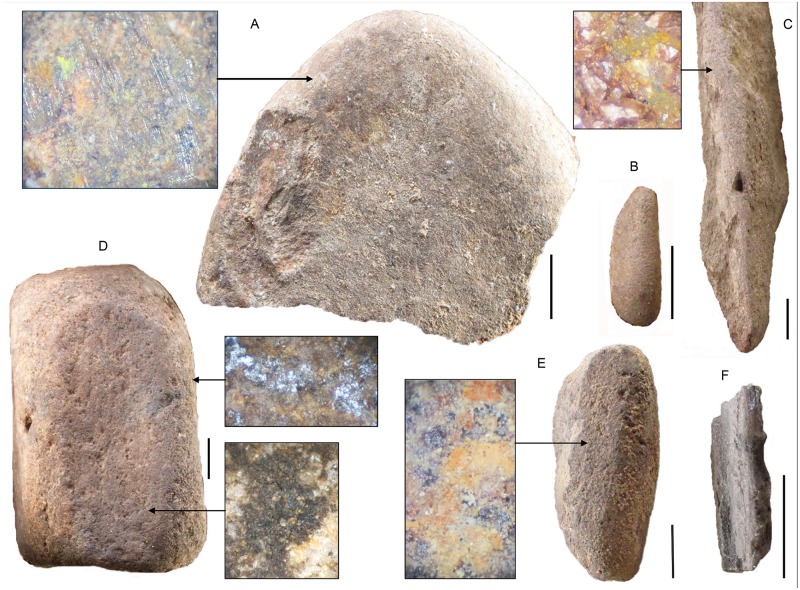
Sibudu grindstones from layers BYA2i to YA. A. Broken round grindstone with enlargement of grinding striations. Square B5a, layer YA. B. Small lozenge-shaped grindstone. Square C5b, Layer YA. C. Straight sandstone slab with ground edge on long axis. The enlargement shows a yellow mineral stain on the ground edge. Square C6c, layer YA2. D. Flat grinding surfaces. The uppermost one shown is concave with a dark brown residue and some black, reflective residue. Square B5c, layer BYA. E. Faceted grindstone with residue that may be crushed bone. Square B5d, layer BYA2. F. Straight sandstone slab with ground edge on long axis. Square C4a, layer BYA. All scales are 10 mm. The residue images are all 45x magnification. All scales 1 cm.

In this layer dolerite recovers its representation (Fig 3) and has equal representation to quartzite and sandstone (autapomorphy). Hornfels disappears among the completed flakes (>2cm), although it is the main retouched rock type (autapomorphy). Regarding blade production there is only a small representation of prismatic blades and bladelet production on quartz, dolerite and hornfels (all of them autapomorphies), probably coming from prismatic cores. There is flake production in quartzite and sandstone. Quartzite bipolar knapping is represented by only four small cores. It is remarkable the presence of a core on flake in dolerite (autapomorphy). Only two retouched flakes occur, one in dolerite and another one in hornfels. A grindstone was made on a faceted pebble (Fig 10).

#### Mottled Grey

This is a really small layer and the main rock type is quartzite (autapomorphy) (Fig 3). Bipolar knapping, bladelet and flake production is represented. There is a big prismatic blade in dolerite and small bladelet fragments in hornfels and quartzite (autapomorphy). The two most remarkable characteristics of the layer are the importance of quartzite and grindstones (autapomorphy). Three grindstones include a round one, a faceted pebble and a grinder with one flat and one round surface (Table 3). There is also a segment with ochre residue which is very similar to GR examples (Fig 9F), this could mean that there was occasional continuity of the hafting techniques (and weaponry uses) between Howiesons Poort and post-Howiesons Poort.

#### YA2

**Fig 11:**
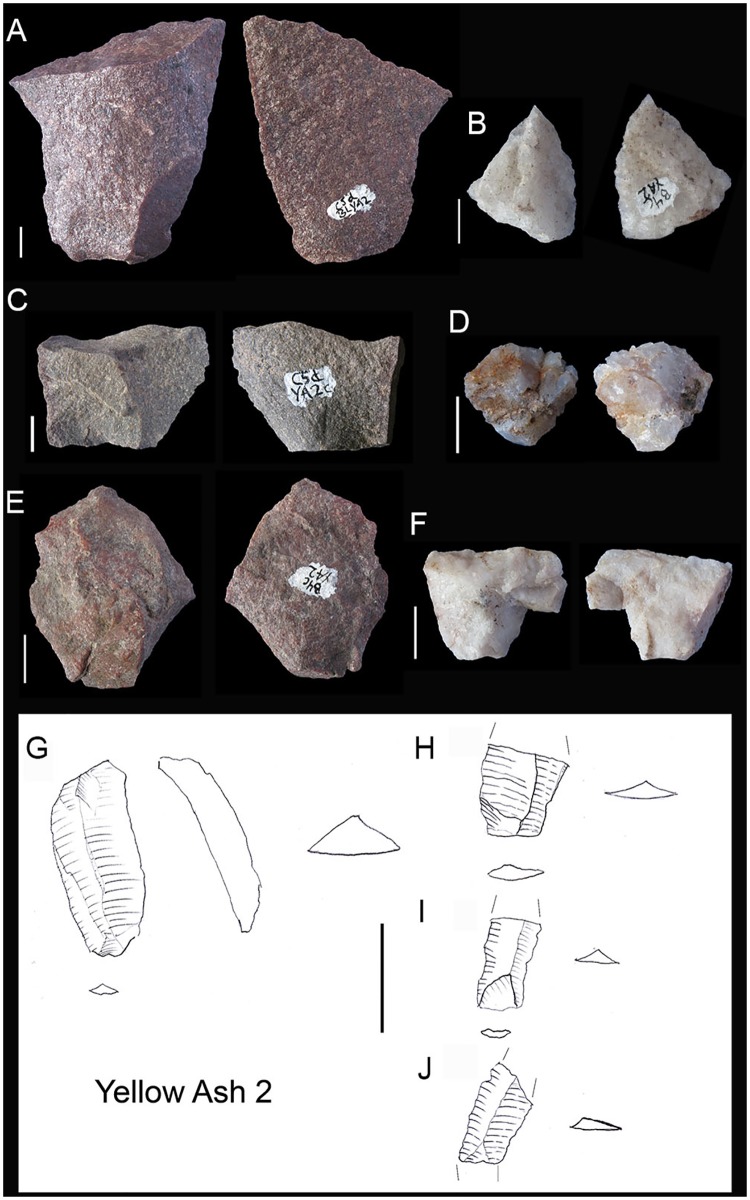
Different blank types in YA2. A, C, E. Quartzite flakes. All scales 1 cm. B, D and F. Quartz flakes. G, H, I, J. Dolerite blades. Scale 3 cm.

In this layer the most important rock types are dolerite, quartz and quartzite (Fig 3). Some of the cortical flakes in dolerite and quartzite show that they were river pebbles. Besides this, there is a great variety of other rock types which have been knapped on the site and they gain importance, such as: andesite, amphibolite, gneiss, crystal quartz, cryptocrystalline material and even knapped hematite. Blade and bladelet production is in dolerite (Fig 11) and hornfels. However, once again, flake production is prominent, especially in hornfels (autapomorphy), dolerite, quartzite, sandstone and quartz. The discoidal knapping reduction is well represented by cores and blanks. Quartzite bipolar knapping is present again (Fig 7). One of the features in this layer (Black Lens in Yellow Ash 2) contains big flake production in quartzite (Fig 11). In contrast with the non-retouched pieces, some of the retouched ones were made on hornfels and dolerite and they are big notches or retouched flakes. Moreover, the main retouched blanks are blades (autapomorphy), whereas most unretouched pieces are flakes. This layer yielded three round grindstones and one straight slab with an end ground to form a flat edge (Table 3 and Fig 10).

#### GYA

This is a very small layer. The only two technological qualitative features picked up in the analysis are blade prismatic production in dolerite and some quartz flakes.

#### BYA

The main rock type is again dolerite, followed by quartzite and quartz (Fig 3). Once again unusual rock types such as amphibolite occur. The blade production is on quartz, hornfels (autapomorphy) and dolerite, whereas bladelet production is on quartz (freehand) and quartzite. Flake production exceeds blade production and discoidal variants are made on quartz, dolerite and quartzite. Quartzite flakes have many Siret fractures, probably due to the use of a hard mineral hammer. There are only two bipolar cores in quartzite. There are two bifacial quartz pieces (similar to the Howiesons Poort bifacial quartz points and the ones in BYA2 i) (Fig 8G and 8H). Grindstones of various types (autapomorphy) are the technological highlight of this layer. They include two round, two faceted, one flat and one thin, straight sandstone slab with a ground edge (Table 3 and Fig 10).

#### YA

The main rock type is again dolerite, followed by quartz and quartzite (Fig 3). Smoky quartz and crystal quartz (autapomorphy) are the main quartz types. In this layer the blade production is on dolerite, hornfels, quartzite and sandstone. There are some dolerite and hornfels bladelets. Discoidal flake production is on quartz, hornfels, quartzite and dolerite, and there are quite typical discoidal blanks (autapomorphies) and one dolerite discoidal core. The dominance of centripetal scar patterns in dolerite also reinforces the importance of flake production (autapomorphy). The absence of bipolar knapping is noteworthy (autapomorphy). Some knapping began in situ because there are several cortical flakes in quartz and quartzite. Three of the four retouched pieces are on hornfels. A lozenge grindstone and a ground, faceted pebble occur (Table 3 and Fig 10).

### Evolved technological characters from GR to YA (Synapomorphies)

In this section we describe the main lithic technology characteristics that show synapomorphies. By this we mean the technological variations that are grouping these layers once ‘new characters’ appear in the succession of layers (see S1 and S2 Tables) and the different nodes in the Tree 1 shown in Fig 2.

Node 15- Differences between GR and the eight uppermost layers

The most relevant changes are the synapomorphies of node 15 (see cladogram tree 1 in Fig 2 and S2 File), because those show all the derived characters that distinguish GR from the uppermost layers (RB to YA).

The most remarkable technological changes are linked to the management of quartz (the disappearance in the upper layers of crystal quartz for knapping and the disappearance of quartz bipolar knapping) and the differences in blade/bladelet production (the disappearance of blade cores and core-related by-products). However, the difference in blade/bladelet production is not because blade and bladelet production disappears, but because the cores and the core-related by-products disappear (such as the semicrests). A most notable change regarding blade/bladelet production is the disappearance of core-on-flakes (with the exception of RB and BYA2) and the so-called ‘Howiesons Poort cores’ (for the definition see [65]). Concerning flake production, this was already present in Howiesons Poort, but what is a novelty is the appearance in all of these layers of quartzite flake production that was completely absent during the Howiesons Poort layers. Finally, regarding this node, we must point out the differences concerning retouched lithics/morphotypes; in seven of these eight uppermost layers (the exception is YA2), the preferred blanks for retouch are flakes (rather than blades) and backed pieces are no longer the dominant morphotype, being replaced by low percentages of retouched pieces with a notable lack of standardization compared with those in the Howiesons Poort layers.

Node 16-BYA/BYA2i

The main characteristics highlighted by the synapomorphies are quartz (the second most important rock), the dominance of centripetal/subcentripal scar patterns for flakes, and the presence of big notches amongst the retouched tools.

Node 13-YA, YA2, RB, Mottled Grey, BYA2

Most of the synapomorphies in this group show absences: such as the absence of bladelets and bifacial pieces.

Node 18-YA-YA2

The synapomorphies in this case are the presence of discoidal flake cores and bladelet cores in dolerite and the main retouched type in dolerite.

Node 17 Mottled Grey/BYA2

This group has two remarkable synapomorphies related to rock types: the dominance of dolerite as the main rock type and quartzite as the second one.

### Overview of the qualitative characteristics

All layers RB to YA show a notable change in terms of representation of rock types compared to GR. This has already been shown by previous technological studies [18, 19, 31, 32], although this change is not abrupt as can be seen with the representation of rock types in RB (Fig 3). Moreover, there are notable differences in the representation of rock types in layers RB to YA. The general trend is the loss of importance of hornfels (although it remains the rock most used for retouched pieces) and the increase of quartz and quartzite (depending on the layers). Furthermore, other rock types (such as andesite, amphibolite and gneiss) that were not present at all in the Howiesons Poort layers appear in some of the younger layers, and these rocks suggest an important change in exploitation of resources.

**Fig 12:**
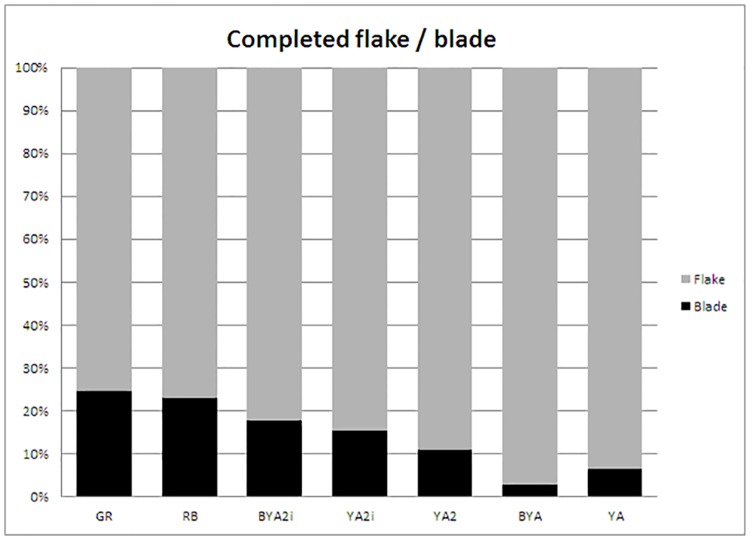
Percentage of completed flakes and blades per layer from GR to YA.

Another major change is the representation of morphotypes and the presence/absence of technological standardization. In GR retouched blanks are an important component of the non-chip sample, whereas in all the uppermost layers (from RB to YA) there is a lower percentage of these pieces (see retouched pieces per litre column in Table 2) and, more importantly, they seem highly unstandardized, with ‘retouched flakes’ being the most common category (Table 2). Indeed, one of the principal synapomorphies in Node 15 is also quite telling: most of the retouched pieces from RB upwards have predominantly flake blanks, whereas in the Howiesons Poort layers, blades were the predominant blank. On the contrary, and surprisingly, the representation of complete unretouched flakes and blades do not change drastically from GR to YA and there is a gradual reduction of blade blanks from GR to YA (Fig 12).

**Fig 13:**
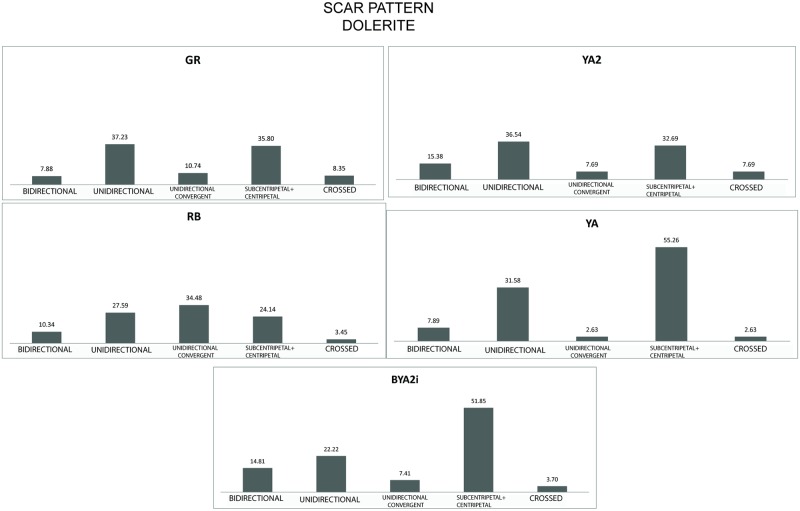
Scar pattern for dolerite in GR, RB, YA2, YA and BYA2i.

Another interesting observation concerns the fluctuating density of lithics per litre. In addition, as shown by the autapomorphies and the synapomorphies (S2 File), the layers overlying GR demonstrate constant fluctuation in technological trends; characteristics appear and disappear. One example of this fluctuation is the dorsal scar pattern for dolerite (the rock type most evenly represented within the different layers analyzed). As can be seen in Fig 13 layers such as YA2 have dorsal scar patterns almost identical those in GR, whereas others such as YA or BYA2i reveal patterns with a dominance of centripetal/subcentripetal scars, probably related to greater importance of flaking knapping methods.

Three technological characteristics are unique to GR: the bipolar knapping in quartz, the *Levallois* flake production in dolerite and the so-called “Howiesons Poort core” blade/bladelet production on hornfels and dolerite. Conversely, some technological trends seem specific to layers RB to YA: freehand knapping of quartzite, several types of grindstone (particularly in BYA, Table 3 and Fig 10) and the lack of standardization of the retouched pieces.

Besides this, and maybe one of the most important conclusions from the descriptive analysis, is that there is a change in the ‘weight’ that certain knapping methods have in GR compared to the uppermost layers (RB-YA). Some reduction sequences that were dominant in GR still appear in the overlying layers (RB to YA), but they seem less well represented than in GR; whereas other technological strategies that were rare in GR seem much more visible in the overlying layers. Maybe the most important technological strategy in this regard is flaking reduction sequences. They were already present in GR, but in all the RB to YA layers they are much more visible (including, as a novelty, quartzite flake production, a synapomorphy); whereas blade and bladelet production were abundant in the Howiesons Poort, but they became rare later and were very seldom represented by the cores and the core related by products. It must be stressed that they do not completely disappear (see for example Figs 4, 6 and 11). Indeed, this could be due to the fact that there was a different intra-site organization regarding blade production. For example blade prismatic production is represented by some blanks in dolerite and hornfels in all the layers overlying GR, but in those layers there are no cores and virtually no core related by-products, which implies that these blanks were part of the tool kit, although they were not produced at the site anymore. In the same manner, flake production in quartz certainly was performed during Howiesons Poort (in GR constitute autapomorphies), as thick quartz flakes were the blanks necessary to produce bifacial points during GR and GS [64], but they were not produced on the site, whereas in the overlying layers the quartz flakes were produced on site (Figs 6 and 11) and they also served as blanks for bifacial pieces (BYA, YA2i, BYA2i).

Another example is the quartzite bipolar knapping. It was already documented in GR, but in the overlying layers it becomes a recurrent strategy, having a peak in representation in BYA2i (Table 1 and Fig 7). The quartzite implemented in this technological reduction has quite a coarse grain, which might imply a very specific functional strategy. Interestingly, bipolar products have low frequencies in some of the upper layers and high frequencies in others. This implies particular uses for the bipolar products and, further, the activities involving their use were intermittent through time. We mention this issue again shortly.

Also, bifacial point production does not disappear completely after GR, and it continues in BYA2i, BYA and YA2i (as also pointed out by Will and Conard [19], although they found them in RB, LBYA, BYA2i and YA).

In addition, it is remarkable that in layers BYA2i-YA there are several grindstones (Table 3 and Fig 10), a tool type rarely represented in the Middle Stone Age. The largest number of grindstones is in layer BYA (n = 6), but layers YA2, BYA and YA have 12 of the 18 grindstones. While the majority of grindstones are round or faceted pebbles, there are oval (lozenge) shapes and an interesting tabular form with a flat, ground edge on the long axis. This last form was not found elsewhere at Sibudu, though it must be said that grindstones are generally rare at the site.

All these examples show that there is considerable change through time in the representation of certain technological strategies. This might be less about change to the technological tradition and more about a change in activities at the site as well as intra-site organization. In other words, layers RB-YA maybe represent short occupations that practice different economic tasks and/or group organizational strategies from those in GR. The technology does not radically change between GR and the other layers; it is the emphasis on certain strategies that seems to change. This emphasis on varied knapping methods appears together with different management of space and tasks at the site: massive bedding units were regularly burnt, abundant domestic fires were burnt, and varied grinding activities took place, together with preferences for new types of ochre and rocks.

### Some typometrical remarks regarding the lithics

Regarding the typometrical characteristics, we have found various issues noteworthy and these are independent of the technological analysis, which is mainly qualitative.

**Fig 14:**
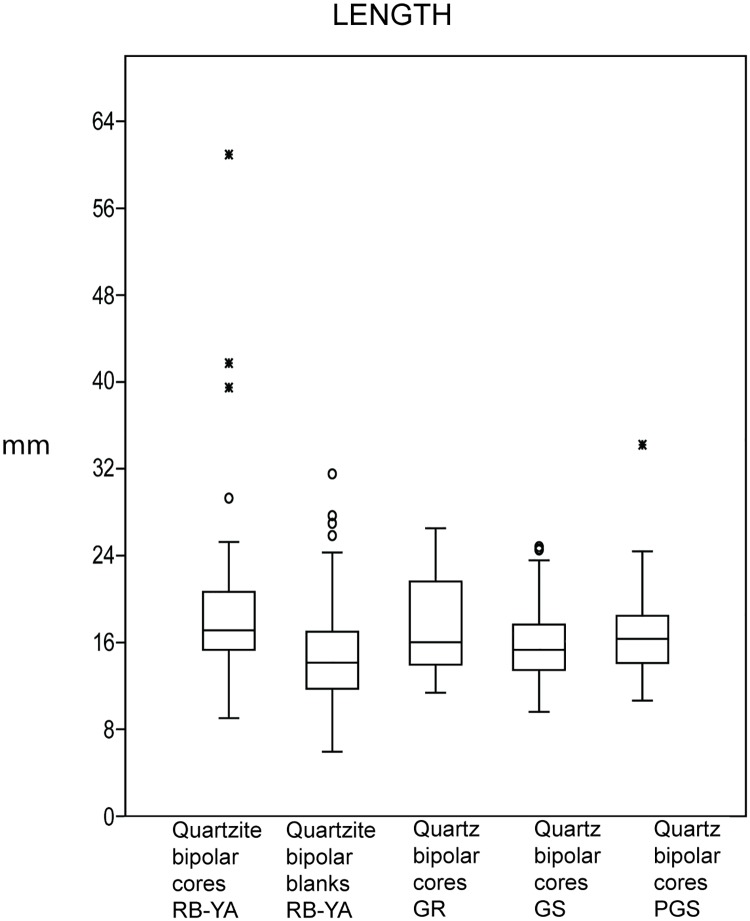
Comparison of the length of bipolar cores in quartzite (RB-YA), bipolar quartzite blanks (RB-YA), and quartz bipolar cores in PGS, GS and GR.

First, we point to the microlithic character of the quartzite bipolar cores and blanks in layers RB to YA. As can be seen in Fig 14, when comparing Howiesons Poort bipolar quartz cores and the freehand quartzite production of these layers, the quartzite bipolar cores and bipolar blanks in layers RB to YA are remarkably small, in most of the cases having lengths less than 20mm. Since this is not a limitation brought about by the available size of the quartzite cobbles (large ones occur close to the site), the quartzite flake production demonstrates a lengthy reduction process (S2 Table). Indeed, the quartzite bipolar cores of layers RB to YA have a median length of 17.12mm and the quartzite bipolar blanks a median length of 14.66mm. The U Mann-Whitney test showed that the length of bipolar cores for RB to YA, GR and PGS is not statistically significant different, whereas for GS it is (See S3 Table).

**Fig 15:**
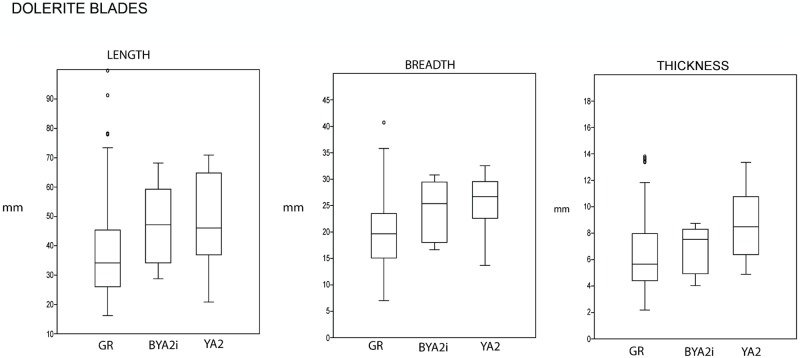
Length, breadth and thickness for all the complete dolerite blades in GR, BYA2i and YA2.

**Fig 16:**
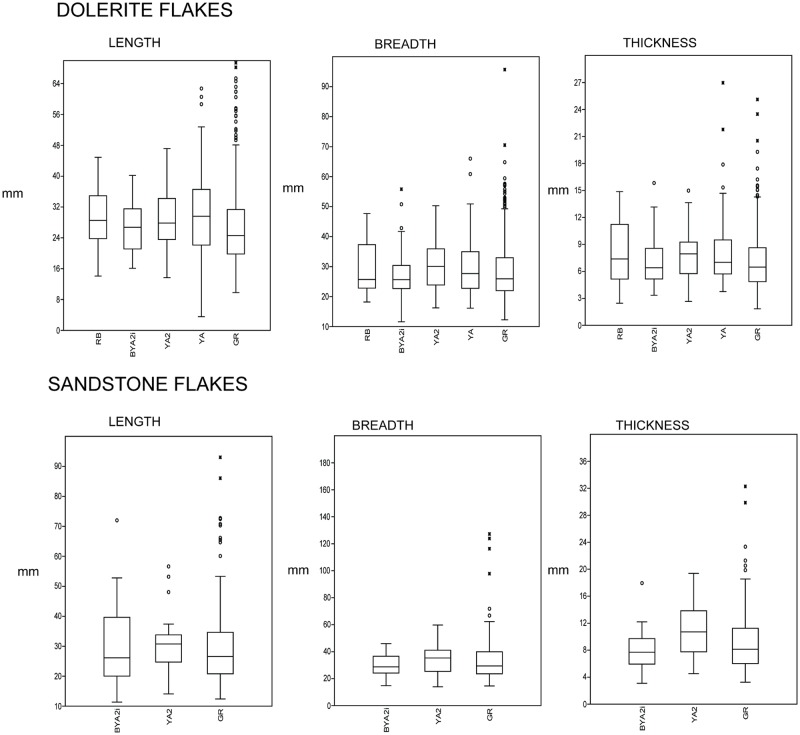
Box-plot of length, breadth and thickness for dolerite (GR,RB, BYA2i, YA2, YA) and sandstone (GR, YA2, BYA2i) flakes.

In regard to the blade production, if we compare the complete blade blanks in dolerite for GR and some of the overlying layers (BYA2i and YA2, which we chose because they were the ones with the most complete blade blanks), it is evident that the blades in BYA2i and YA2 are manifestly bigger than those in GR (see the comparison in Fig 15). Moreover, the difference is statistically significant between GR and YA2 for length, breadth and thickness and between GR and BYA2i for length (S3 Table for normality tests and U-Mann-Whitney tests for all these variables), whereas for the flakes in dolerite this distinction varies between the layers and in some cases is significant (GR-YA; GR-YA2, GR-RB), but in some others there is no difference (GR-BYA, GR-BYA2i) (Fig 16 and S3 Table).

## Discussion

Several previous studies have grappled with descriptions of, and explanations for, the transition from the Howiesons Poort to post-Howiesons Poort at Sibudu [19, 31, 32, 44] and other important sites in southern Africa, such as Rose Cottage Cave [21, 66, 67] and Klein Kliphuis [22]. Papers that have dealt with the transition or with the post-Howiesons Poort more generally are the following: [16, 22, 68, 69]. Moreover, Howiesons Poort has attracted special attention because of its putatively idiosyncratic technology, sometimes even called precocious and linked to symbolic behaviour [70, 71]. For a recent synthesis of different hypotheses regarding its appearance and disappearance see Dusseldorp [72]; he explains the transition as the result of changes in both resource availability and mobility strategies. Arguments that have focused on environmental explanations for the transition include, for example, Deacon [71], Ambrose and Lorenz [73] and McCall [74], among others. In other words, previous explanations for the appearance and disappearance of Howiesons Poort have concentrated on its characteristic material culture as a particular environmental adaptation in southern Africa to particularly harsh Late Pleistocene conditions. This argument has been criticised (for example [34]) because the Howiesons Poort is found in many different environmental niches in southern Africa, so it is clearly not an adaptation to one of them. Others proposed that Howiesons Poort could be attributed to migration or the technology of a hominin group that arrived in southern Africa, then disappeared again [7, 75], whereas Volman [76] saw it as a change in fashions.

Specifically regarding Sibudu Cave, Cochrane [31, 32] was the first to tackle the issue of the disappearance of Howiesons Poort and the beginning of the post-Howiesons Poort. His study was made on a relatively small sample of lithics from a single square, B5, and his conclusions must be viewed as insightful in this context. He inferred that in a brief period after the Howiesons Poort ‘contingent factors disrupted the normal pattern of raw material usage’ and during this time quartz and quartzite were extensively exploited [31]: 86. Later, in a different synthesis of the same material, Cochrane [32] pointed out how the deepest layers attributed to post-Howiesons Poort contained discoidal and Levallois flakes, bipolar knapping and only rare retouched pieces. His conclusion was that post-Howiesons Poort occupations of Sibudu were organized differently from earlier Howiesons Poort ones. He argued that an abrupt technological change occurred between the Howiesons Poort and post-Howiesons Poort. This is in contrast to the sequence from sites such as Rose Cottage, where a more gradual transition was proposed [21, 66, 67]. One of Cochrane’s [31, 32] most noteworthy observations was the abrupt change in terms of rock selection and use.

In our analysis of the Sibudu assemblages, which is advantaged by access to lithics from six squares, whereas Cochrane had only one, we have seen that the change in rock and mineral use is not as abrupt as was first thought (Fig 3). For example, RB has a very similar raw material distribution to GR whereas its overall technological characteristics connect it more to the overlying layers (Fig 2). Moreover, if all the rock types are considered separately, we see that there is a notable fluctuation between the different rock and mineral types represented and that quartz is not always the predominant material in the layers immediately overlying the Howiesons Poort (*contra* Cochrane [31, 32]). Cochrane was dealing with a small sample, so he combined quartz and quartzite into one category, and hornfels and dolerite into another, and discounted sandstone. Besides this, it is noteworthy that knapping methods including discoidal and *Levallois* flaking were documented in GR [20]. Also the presence of quartz bipolar knapping is a constant microlithic strategy in the Howiesons Poort layers [63], but not in the immediately overlying layers where the quartz knapping is exclusively freehand and the microlithic strategy of bipolar knapping was only on quartzite (Fig 7).

A recent technological analysis of Sibudu’s layers RB to G1 by Will and Conard [19] highlighted the abundant diachronic variability of these layers overlying the Howiesons Poort. Moreover, they point out how all these layers and the overlying ones with a ~58 ka age show ‘short-term technological and typological changes’, often a ‘gradual, incremental and accumulative nature likely reflecting intergenerational transmission of information’ [19]. This study reinforces the idea in previous works by the same team that the layers overlying Howiesons Poort demonstrate great variability in raw material provisioning, reduction sequences, techniques, and tool manufacture and use [18, 77, 78]. In the 2016 study these oldest post- Howiesons Poort layers (RB-G1) are attributed to an early phase of the ‘Sibudan’ [19]. Younger Sibudan layers are characterized by: fragment procurement and use of dolerite and hornfels, multiple core reduction methods, variety of blank types including convergent flakes and blades, predominant production of flakes by hard hammer percussion and abundant unifacial points (particularly in its upper part: BM to BSP layers). The authors, after this last work, subdivided the Sibudan into four different facies, with layers RB to G1 being the oldest one. They characterized this oldest facies by: its focus on ‘local raw materials’ (dolerite, sandstone, quartz and quartzite), low frequencies of retouched pieces, less regular production of unifacial points in comparison to overlaying layers, and the absence of Tongatis and Ndwedwes (see [18] for the techno functional definition of these lithic categories). Finally, they claim that the only direct technological link to the Howiesons Poort is the presence of quartz bifacial pieces similar to the ones found in Grey Rocky (GR) and Grey Sand (GS) [64].

In contrast, our comparison of lithics from GR (attributed to Howiesons Poort, see [20]) and those from the overlying layers, RB to YA, suggests that there are several technological links between the older and younger assemblages. For example, the flaking methods that we described in detail for GR in a previous work [20] increase in the overlying layers (such as BYA2i or YA, which have clearly discoidal reduction methods). Moreover, prismatic blade production (evident in blanks and core-related by-products) continues in the layers overlying GR (see Figs 4, 6 and 11), but is represented to a lesser extent. Another example of technological continuity is quartzite bipolar knapping to produce small flakes and bladelets. This was already present in GR [20, 63] and it continues in the overlying layers with a notable peak BYA2i (Table 1). Layer RB is a good example of mixed technological characteristics because it has, on the one hand, a very similar rock type representation to GR, continued blade prismatic production and even core on flake production (Fig 5A). On the other hand, the synapomorphies show clearly how this layer is technologically more closely related to the layers overlying GR (Fig 2). Concerning the retouched pieces, not only bifacial pieces appear in both GR [64] and the RB-YA layers ([31]: 162, [19]) (Fig 8), but also a few backed retouched tools. These are not abruptly abandoned in layers RB-YA because there is a segment in each of the layers RB, BYA2i and Mottled Grey under YA2 (Fig 9B, 9D and 9F). The appearance in layers YA and YA2 of bone tool manufacture in the form of a *pièce esquillée* and a bone retoucher [10] is further evidence of technological continuity between the Howiesons Poort and later occupations, even though the tradition of bone tool manufacture was infrequently activated after the Howiesons Poort. Both bone tool classes were present in the Howiesons Poort. In other words, bifacial pieces should not be considered the sole trait that links GR technologically with the immediate overlying layers (*contra* [19]).

Our study of technology leads us to a conclusion that we have previously proposed: there is not a complete disappearance, but a rearrangement of technological strategies between GR and the overlying layers. Some technological strategies that are present yet uncommon in GR become abundant in the overlying layers, whereas others that were fundamental to the Howiesons Poort in GR continue in the overlying layers, but are not well represented. We can add to this that lithic technological strategies appear and disappear as pulses in the younger RB-YA layers. In other words, technological change does not seem cumulative in the layers from RB to YA, but rather pulsating.

Notwithstanding the technological continuities listed, there is notable change (explained extensively above by all the synapomorphies of node 15 of our cladogram) between layer GR and layers RB to YA. Indeed, among the most notable changes in these younger layers we stress three technological strategies, which have implications for our discussion:

- The importance of flake production from a discoidal knapping method in layers RB to YA.
- The unstandardized retouched pieces and the much lower representation of retouched pieces than in the Howiesons Poort.
- The higher representation of grindstones than elsewhere in the sequence because of the increased importance of specific extractive processes at the site.

**Fig 17:**
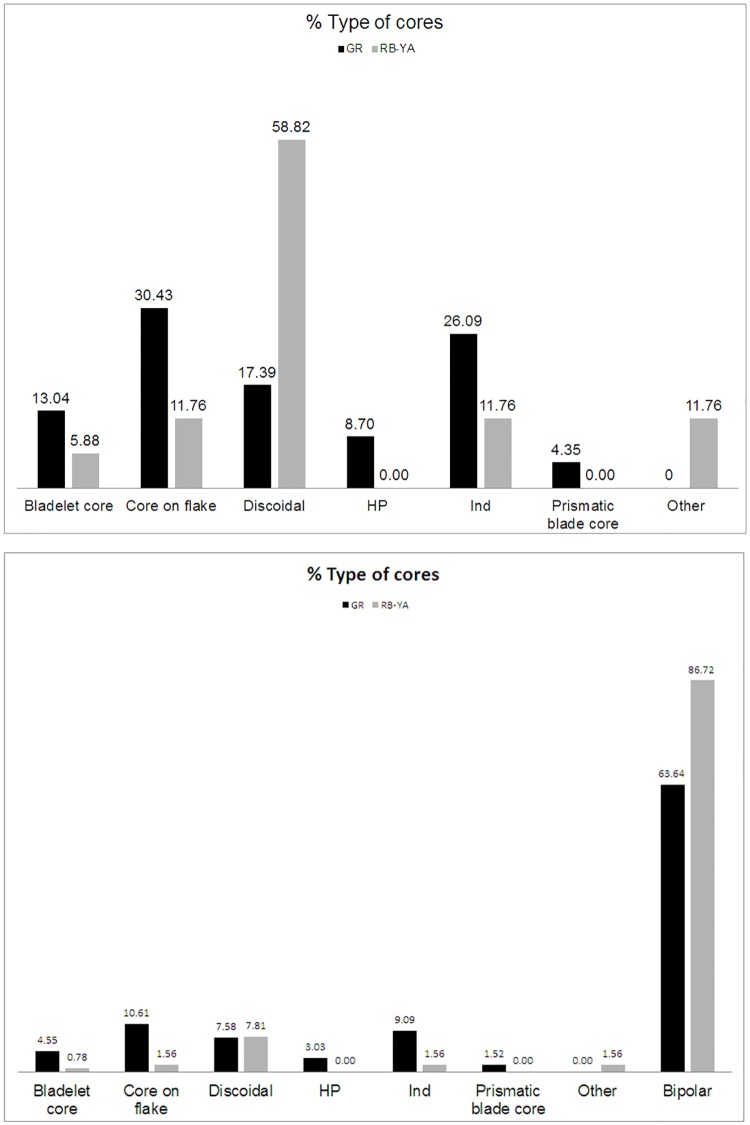
Percentages of cores in GR and RB-YA. Top: without bipolar cores. Bottom: with bipolar cores (data from Table 5).

**Fig 18:**
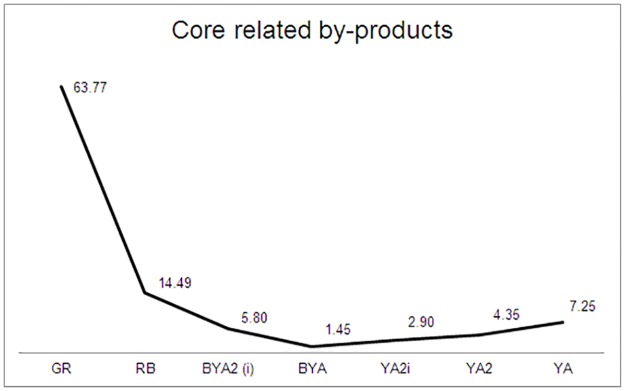
Percentage of core related by-products for GR and RB-YA layers.

The relevance of flake production (including the quartzite discoidal method as a novelty in the RB-YA layers) is that it points to the importance of non-retouched thick flakes in local raw materials (such as sandstone, quartzite, dolerite and quartz). This could imply a functional shift that developed on the site after the GR occupation. It is also clear that in these layers what it has been called ‘expedient core technology’ in the literature [30, 79] becomes important. In this regard we are referring particularly to discoidal cores (Figs 5 and 17) which have increased representation in layers RB to YA in comparison to more formalized cores, such as the so called ‘Howiesons Poort cores’ or prismatic blade cores, well represented in the Howiesons Poort layers [20]. Moreover, the notable drop in layer BYA2i to YA of core related by-products from freehand knapping methods provides further support for the argument that knapping is not as formalized as in the Howiesons Poort (Fig 18). This important change is accompanied by bipolar knapping in some of these layers (Tables 1 and 5 and Figs 7 and 17 below). Bipolar knapping has been described as an ‘expedient strategy’ in other studies [30, 79]. However, it must be borne in mind that bipolar reduction strategies were already abundantly present in the Howiesons Poort layers (see previous works on this issue [63] and Figs 7 and 17 and Table 5), thus, this is a signal of continuity between GR and RB-YA, and we believe that these tools might be reflecting different functional strategies that are not possible to elucidate without use-wear analysis of the blanks. We suspect that the quartzite bipolar blanks of layers RB to YA might be used for tasks different from the quartz ones that are common in the Howiesons Poort layers, but clearly this suggestion must be tested. The abundance of quartzite bipolar knapping products in layers such as BYA2i is indicating once again the rapidity of the knapping method. Finally, the importance of this strategy at Sibudu is also demonstrating that the microlithic blanks produced with this knapping method are not an exception in Middle Stone Age assemblages, nor are they a technological marker for the Later Stone Age [63] *contra* [14, 80].

**Table 5:** Types of cores per layer from GR-YA.

	GR		RB-YA	
	N	%	N	%
Bladelet core	3	4.55	1	0.78
Core on flake	7	10.61	2	1.56
Discoidal	5	7.58	10	7.81
Howiesons Poort core	2	3.03	0	0.00
Indeterminate core	6	9.09	2	1.56
Prismatic blade core	1	1.52	0	0.00
Other (tested cores)	0	0.00	2	1.56
Bipolar	42	63.64	111	86.72
**Total**	**66**	**100**	**128**	**100**

Besides this, the lack of standardization of the retouched blanks in BYA2i to YA is also a major behavioural change in the sense that it might be pointing to a change in mobility strategies that we shall discuss later.

The 18 grindstones or grindstone fragments from layers BYA2i to YA incorporate several morphological forms and were probably used for grinding or smoothing a variety of materials, or perhaps the same materials in a different way. Bone and other animal products were observed by Williamson on a small sample of grindstones [81]. Our new study confirms this observation on a larger number of grindstones, but we have also detected some residues of a resinous substance and a few traces of red and yellow ochre. Certainly resin and ochre occur regularly in the Sibudu sequence, but they seem to have been processed in a new way in layers BYA2i to YA. Thus it seems likely that these grindstones were used for various extractive tasks yet, surprisingly, plant processing does not seem to be one of these activities.

These important changes coalesce after a probable hiatus of approximately 4000 years between the Howiesons Poort and the next occupation.

As previously stated, all these examples demonstrate change in the representation of certain technological strategies, which could mean a change in functional activities at the site as well as intra site organization from layer RB onwards. Indeed, this group of 58 ka old layers may represent multiple occupations that developed somewhat different regional mobility, subsistence tasks and site organization compared to the ones in GR. Nonetheless, there may not necessarily have been a radically abrupt change and abandonment of Howiesons Poort’s lithic strategies as pointed out above and as pointed out in some previous analyses at other sites [82]. Thus, all the technological links between GR and the overlying layers should be considered as a gradual, not immediate, abandonment of the Howiesons Poort technocomplex at Sibudu.

The final question that we add to this discussion is: why were there shifts in lithic economy and site management at Sibudu from 62 to 58 ka ago? We examine four hypotheses which are not mutually exclusive: environmental change, changed subsistence strategies, a change of behaviour to reduce risk (in terms of subsistence) and a change in settlement pattern that resulted in decreased group mobility.

First, we examine environmental reasons. The micromorphology and mineralogy studies do suggest some change, in particular that from slighter moister conditions in the Howiesons Poort to drier ones in the subsequent occupations [35]. This trend was corroborated by a CA_GIS_ study of seeds and charcoal [83]. The analysis shows that during the Howiesons Poort winters were slightly colder and drier than present, whereas summer conditions were rather like those of today. Post-Howiesons Poort winters were also colder and drier than present, but presumably colder than during the Howiesons Poort. Thus the evidence from Sibudu suggests not only that there were very slight environmental shifts between the Howiesons Poort and post- Howiesons Poort, but that the MIS4 and MIS3 environments were not unlike those in the area today.

Charcoal analysis adds an interesting dimension. The Sibudu charcoal is anthropogenic in origin and has most often been recovered from combustion features. We assume that the firewood was selected for its burning properties and that people collected wood with the attributes required for specific heating purposes [84]. *Podocarpus* (Yellowwood) sp., occurring in moist evergreen forests, is the dominant taxon in the GR and GS Howiesons Poort layers [41]. However, in BYA2i wood from the evergreen forest persists, but is supplemented by wood from a mosaic of vegetation types. The charcoal analysis suggests that there was high diversity of woody species in the early ~58 ka occupations, compared with lesser diversity in the Howiesons Poort [84]. Archer’s [85] ethnographic study in Namaqualand suggests that people walk two to three kilometres to fetch the fire wood they desire, so, if similar subsistence strategies were in place at Sibudu, the varied vegetation communities (forest, savanna, riverine, thicket and grassland) are likely to have been within a few kilometres of Sibudu whereas they were probably farther away during the Howiesons Poort. In the Howiesons Poort swathes of evergreen forest were probably more pervasive and people would have had to walk farther to reach savanna and grassland so they may have relied more on the evergreen forest close to the site for the provision of their firewood and perhaps their meat supply.

Regarding the link between faunal remains and local environments in the Howiesons Poort and post-Howiesons Poort layers, Clark and Plug [44], concluded that layers YA2 to G1 in the early post-Howiesons Poort (that they named post-Howiesons Poort MSA 2) contain faunal remains predominantly from species that could be expected to occur in the riverine forest around the uThongathi River. The post-Howiesons Poort MSA2 shows an intermediate pattern between the Howiesons Poort (largest proportion of species that prefer closed or semi-closed environments such as vervet monkey, blue duiker, red duiker, bushbuck, bushpig, and the banded mongoose) and the post-Howiesons Poort MSA 1 (P1 to BSp) with large grazers such as the blue wildebeest, hartebeest, and zebra suggesting more open environments. In other words, the faunal remains demonstrate a prominent change in the sequence only more recently than the period we deal with, that is in the so-called post-Howiesons Poort MSA1 (P1 to BSp).

Herries [86] conducted a palaeomagnetic study for the post-Howiesons Poort layers of Sibudu where he proposed that the lower layers (YA2 to G1) have a different signal from the upper layers (P1-BSp). Indeed he proposes that the lower layers of the post-Howiesons Poort probably correspond to the cool MIS 4 while the layer P1 probably represents the transition to a warmer MIS 3.

Using these proxies, it seems that between the Howiesons Poort and the post- Howiesons Poort analyzed here there are some gradual local paleoenvironmental changes. Moreover, a noteworthy change is only recognised through faunal and paleomagnetic analyses in layers younger than the ones we analyzed [44, 86].

The micromorphology, the charcoal analyses, and the faunal remains are pointing to small local environmental changes which differ from those recorded in other areas of South Africa between the Howiesons Poort and the post-Howiesons Poort. As just explained using fauna and palaeomagnetism, important environmental change only occurs higher up in the sequence in what Clark and Plug [44] called post-Howiesons Poort MSA 1 (P1 to BSp). Therefore, we do not think that the technological change we see at Sibudu is driven by climate change, and the vegetation mosaic referred to here is a specifically local phenomenon. In the broader southern African context, Jacobs and colleagues [34] observed that Howiesons Poort sites crosscut different habitats, from forest to coast to arid lands. Consequently, climatic and habitat shifts seem an unlikely cause for change in this technology [87].

As other authors have pointed out, technology can inform indirectly about subsistence strategies [26, 27]. The fact that backed implements almost disappear in the RB to YA layers, and that such tools have been attributed by various analysts to hunting strategies at Sibudu [2,3], might be suggesting that there is a drastic shift toward a completely different economy in these layers, maybe towards higher exploitation of plant resources. As Torrence points out ‘the pursuit of immobile resources [such as plants] means that constraints placed on technology as a result of demands for scheduling activities are reduced’ [26]:21. This working hypothesis, that plant resources were more favoured in the activities developed at the site in these 58 ka layers, could partially explain the unstandardized tool kit and the low frequencies of formal tools from RB onwards. However, we do not have data to sustain this hypothesis; even though organic preservation is good, there is no evidence for greater plant food exploitation, and underground storage organs are absent from Sibudu.

The third hypothesis is the reduction of short term risk, also pointed out theoretically by Torrence [27]. It is usually accompanied by a reduction or simplification of technology. For Torrence [27] risk, in term of subsistence, is understood as the probability of failing to meet dietary requirements. Following this, the severity of risk (in terms of subsistence) will determine the extent to which technology needs to be reliable. Therefore, a complex technological system will normally be related to higher risk environments and vice versa. In other words, when risk is lower the need to maintain reliable complex tools which guarantee subsistence partially disappears. Torrence used Oswalt’s [28] distinctions of tools in order to evaluate the degree of complexity of a tool assemblage. Perreault and colleagues [29] have a similar approach to Torrence and they also used Oswalt’s [28] ideas to study tools: they suggested that the complexity of a technology can be measured by counting the number of “procedural units”, in the form of manufacturing steps that result in the finished technological product. Counting procedural steps for actions (and expressing them in cognigrams) was developed by Haidle [88]. Core preparation techniques, blank production techniques, product shaping and core rejuvenation are examples of this [29]. At Sibudu there is some evidence to support this hypothesis (Tables 2 and 5 and Figs 5, 7, 17 and 18). Although we have not formally applied this approach to our study, after the technological analysis it is clear that there are fewer procedural units involved in the production of the RB–YA assemblages than the GR one. As previously stated, one of the main changes in the younger occupations is the manifest lack of standardization of retouched pieces (‘retouched flakes’ being the most abundant tool). Besides, the dominance of discoidal and bipolar knapping as the main reduction strategies highlights the relevance of what might be called ‘expedient core technologies’. In the light of the technological analysis presented here, changes in short term risk could have contributed to the behavioural change detected at Sibudu for the period analyzed. The different technological synapomorphies are pointing towards a general, more expedient technology from RB upwards in the sequence, implying low risk on the Torrence model. However, we should ask why there should be lower subsistence risk in the period studied here? Drastic environmental change can be a catalyst, but we have already eliminated that possibility at Sibudu. Another possibility is innovative management of the spatial and temporal distribution of resources (as has been claimed in Australian assemblages where there was a shift towards more expedient technologies, see for example Lourandos [89]). Another way of reducing risk is through the invention of new ways of managing resources. However, with the evidence at hand, this is highly speculative, because apart from different spatial management through new waste disposal methods (see below discussion on micromorphology) there is no other functional evidence to support this suggestion.

The fourth hypothesis proposes a change in settlement pattern. Parry and Kelly [30] noted from some North American archaeological examples (together with ethnographic examples) that in different types of societies the change from a curated technology towards a more expedient technology can come about alongside a shift in settlement pattern. Their main argument is that expedient core technologies usually correlate with decreased residential mobility when raw material is abundant near the residential site. The examples they present are societies that developed sedentary patterns, but residentially mobile hunter-gatherers are mentioned (such as the Ngatatjara and Pintupi of Western Australia [90, 91], the Xeta of Brazil [92] and the Tjimba of Namibia [93]). One of their main arguments is that mobility plays an important role in determining the composition of lithic tool kits. Stone tools are usually heavy to carry. It must be borne in mind that the tools or the raw materials needed during a journey sometimes cannot be anticipated. Bifacial technology or highly standardized technologies can be extremely useful as multi-task tools and they can also be repeatedly reused. Moreover, in the case of highly formalized shapes they are usually easy for transportation and easy to replace for groups that require a highly mobile strategy through the landscape. On the contrary, good portable tools are not as essential for many sedentary societies that do not rely only on the resources obtained using the toolkit. They also point out that expedient core technology strategies can be found among mobile hunter- gatherers, but these are usually hunter-gatherers who have abundant knappable rocks available. Moreover, the technique could be implying (as it is the case among some tropical huntergatherer societies) that new organic tools are becoming more important in the tool kit, making stone tools less essential. The examples that Parry and Kelly [30] use are societies that shift from mobile hunter-gatherer activities to sedentism. In our case, we do not believe that such a drastic change occurred at Sibudu, but certainly different strands of evidence (see below) indirectly imply a shift towards a decrease in mobility patterns that in the long term probably had an impact on the organization of the tool kit described in this paper.

For the case study analyzed in this paper we should ask: What is the evidence at Sibudu for reduced residential mobility at ~58 ka ago? Could it explain the patterns we observe at the site at this time?

We suggest that several strands of evidence together with the lithic technology point in this direction: the deep stratigraphy that built up in a relatively short space of time perhaps mostly because of repeated burning of plant bedding and waste bone, collection of local rocks for knapping, collection of locally-occurring ochre and the collection of firewood from a variety of vegetation communities that were probably fairly close the site.

We have several times remarked on the depth of the sediments and the fact that the occupations following the Howiesons Poort are characterized by a considerable, rapid accumulation of anthropogenically-derived sediment. The geoarchaeology has shown that in almost all the 58 ka layers mm-scale bedding units built up from monocotyledonous plants that were later burnt, then replaced, and burnt again, so that either fairly continuous habitation of the shelter could take place or it could be re-occupied within a short space of time. Activities were carried out intensively in the bedding areas; both bone and stone were processed here [38]. Since charcoal was also well represented in bedding areas [38], it seems that wood may have been heaped on the bedding to start fires that would ignite it. Once again, such practices might be pointing to mobility strategy that was considerably reduced compared to that of pre-60 ka ago occupations. Thus the stratigraphy implies either more frequent visits to the site than previously, or to repeated, longer-term occupations. The stacks of burnt bedding dating ~58 ka ago tend to support the interpretation of a relatively long-term occupation strategy, but we cannot estimate the time frame of the occupations based on the data we have. Burning fusty sedge and grass bedding would have provided a quick solution to cleaning the site, ridding it of parasites, and creating a means to allow continued, disease-free occupation of the site. If the site had simply been abandoned after short occupations (as may have happened at least some of the time before 58 ka ago) this maintenance strategy would not have been necessary.

Another site management strategy involves the discard of waste bone. The BYA2i to YA layers discussed here have significantly larger proportions of highly burned (calcined) bone, with poor cortical preservation, than either earlier or later layers [84]. While some bone may have been accidentally and post-depositionally burned when buried under hearths or incorporated in bedding, some bone may have been deliberately disposed of in fires as a means of cleaning the site [94].

Ochre use changes also between the Howiesons Poort and overlying layers. Higher percentages of soft, clayey ochre pieces occur in the Howiesons Poort than later, whereas silty ochre was more popular in layers BYA2i to YA. Geological occurrences of silty ochre are close to Sibudu (about one km away) so a preference for this type may signal not so much tasks different from those in the Howiesons Poort, but more of a tendency to collect raw materials close to the home base.

This suggestion is supported by the collection of local rocks for knapping (Fig 3). Sandstone is the roof rock of the shelter, and quartzite can be readily collected on the river banks close to the rock shelter, so reduced mobility is implied when rocks like these become commonly used and when hornfels, which occurs about 20 km from Sibudu, becomes rare.

The charcoal identifications mentioned earlier record higher diversity of woody species in layers BYA2i to YA, compared with GR. People in the later period seem to have made more use of a wide range of vegetation communities for wood collection, and a mosaic of communities may have been closer to the site than previously. Another possibility is that people remained longer at the site than in previous times and had to range more widely for their firewood, thereby collecting from a diversity of plant patches. This practice of firewood collection is supported by the very slight change in hunting patterns; these reflect a shift from targeting predominantly small game dwelling in closed environments to a mixture of small and large plains game (but prey choice was still quite similar to that in the Howiesons Poort based on the identifications by Clark and Plug [44]). The shift to hunting large plains game took place more recently than the period under discussion here.

The volume density of lithics does not demonstrate major differences between the Howiesons Poort and the younger RB-YA layers (Tables 1 and 2) and it is not possible to interpret meaningful settlement differences based on these data. This is particularly true because we are not dealing here with time-averaged assemblages and it is therefore not really valid to compare densities between these sets of layers [95]. The GR and GR2 layer potentially represents several thousands of years and this is in contrast to the BYA2i to YA layers that may have ages just a few hundred years apart. What is worth noting is the difference in volume densities between the alternating yellow ash and brown or grey layers of the RB to YA sequence (and they also form monophyletic groups in our cladistics analysis Fig 2). The yellow ash layers tend to have the lowest densities of lithics, compared to the brown and mottled grey layers with relatively high volume densities of lithics. This suggests a greater anthropogenic contribution to the brown, organic-rich layers than to the less organic-rich yellow ash layers.

Finally, to support this proposed change to low residential mobility, we revisit the three technological main characteristics highlighted in the cladistics analysis and technological analyses. These seem essential to support this last hypothesis: the importance of flake production (mainly from discoidal knapping methods), the unstandardized retouched pieces and the relatively high representation of grindstones. The first two imply that the toolkit is not highly standardized (in contrast to the assemblage with backed pieces in the Howiesons Poort), probably because less group mobility is required. This does not mean that standardized tools completely disappear (Fig 9), but that they are not as essential as they were before. Certainly the dominance of different flaking methods is indicating a drastic change in terms of lithic organization. The virtually disappearance of backed pieces implies that these highly portable and replaceable pieces are not essential anymore and that they can be replaced by other types of stone tools not so well adapted to transport, but easy to make (and then discard) in the surroundings of Sibudu. The grindstones imply extractive activities that might be related to the new pattern of mobility strategies and organization.

In short, our data tend to support the hypothesis of reduced mobility at Sibudu as one explanation for the shift in lithic strategies between the occupations in the Howiesons Poort and those that followed it (at least from layers RB to YA). Environmental factors cannot be convincingly shown to have caused this change. Therefore, what was the trigger for changing to different types of activity that required a less curated technology? We are inclined to favour social impetus after all the evidence reviewed. We do not have enough data to infer the types of social relationships that prevailed either in the Howiesons Poort or later, but there is a possibility that changes in band size and/or fluidity influenced mobility strategies (as argued in [37]). We do not know whether group size was larger in the post-Howiesons Poort than the Howiesons Poort or whether small groups occupied the site for longer.

Our explanation for the appearance of the expedient tool kit at 58 ka in Sibudu may resonate with data from other Middle Stone Age sites, but we cannot be certain of this because we are not in possession of as much archaeological background for other sites. There are, however, some clues to suggest that reduced mobility may also accompany the change from Howiesons Poort to post-Howiesons Poort assemblages at other sites where the transition takes place. At Klipdrift, Reynard et al. [96] consider that the phase that is possibly transitional from Howiesons Poort to post-Howiesons Poort at about 60 ka ago may coincide with lower population densities than previously. In this potentially transitional period, the faunal assemblage is dominated by tortoise and small mammals.

MIS3 occupations are not well represented in the Cape. Marean and colleagues [97] suggest that small populations in the Cape in MIS3 may be one reason for this. Silcrete loses popularity and quartzite dominates lithic assemblages, but there is continued focus on coastal resources, despite the presence of a significant coastal plain [97]. At Pinnacle Point 5–6 (PP5-6) low densities of lithics imply that occupations there may have declined in MIS3 [97].

In a recent paper on PP5-6 Wilkins and colleagues [98] hypothesize “that humans responded to MIS 4 glacial environmental conditions at PP5-6 with increased population or group sizes, ‘place provisioning', longer and/or more intense site occupations, and decreased residential mobility”. The main argument supporting this hypothesis relies on the availability of the raw materials importantly influenced by glacial changes on the coast of Western Cape. According to their interpretation, local raw material availability was potentially less in MIS 4 than MIS 3 because quartzite cobble beaches were no longer rejuvenated by an active coastline, and because groups were occupying the site for longer in MIS 4. For Sibudu we know that the glacial/interglacial climate change did not impact on the raw material availability because, unlike PP5-6, Sibudu was largely unaffected by sea-level changes and the size of the coastal plain. The paleogeography of Sibudu differs from the one in PP and responses to the glacial/interglacial cycles are not uniform across the sub-continent. Moreover, as pointed out before, the paleomagnetic study of Herries [86] at Sibudu actually identified the change between MIS4 and MIS3 high in the post-Howiesons Poort stratigraphic sequence and not in the layers analyzed here, so there does not seem to be a correlation between the timing of the glacial/interglacial change and that of the technology. Furthermore, regarding raw materials, dolerite, quartzite and quartz were probably equally available in different phases of the Pleistocene around Sibudu. As stated before, we suspect that the main trigger for the changes in the toolkit at Sibudu was social (and therefore very difficult to detect). Nonetheless, following previous theoretical and hunter gatherer studies we argue that the shift towards a more expedient technology is pointing to reduced residential mobility in the post-Howiesons Poort and not in the Howiesons Poort as suggested by Wilkins and colleagues [98]. Since Sibudu and PP5-6 occur in completely different local settings and were affected differently by glacial/interglacial shifts, our Sibudu interpretation does not imply that Wilkins and colleagues [97] were wrong in their interpretation at PP5-6.

In 2012, McCall and Thomas [82] proposed that, given the Howiesons Poort technological organization, a settlement system of long-term residential camp occupation, in combination with logistical trips targeting specific resources, would seem consistent with the faunal data from Sibudu. The authors comment, however, that one of the main things missing from their model is evidence for ‘logistical foraging trips’. There still there is no evidence for ‘logistical trips’ in the archaeological record of these Sibudu layers. We think that, with the additional, updated information that we have supplied here, our present hypothesis is stronger than theirs.

One of the characteristics of post-Howiesons Poort assemblages throughout southern Africa is their high variability, at least from a typological point of view (no cross-site technological analysis has yet been undertaken) [11, 16, 17, 68, 78]. The variability suggests, on the one hand, that inter-community contact may have been less than in previous times. On the other hand, the variability may point to a wide variety of tasks being conducted after about 58 ka ago. For example, the Swartkop Hill site, Namaqualand, contains high frequencies of awls although this tool type is generally quite rare in other Middle Stone Age assemblages [99].

In this paper we have assessed lithic technological variability for layers GR (attributed to Howiesons Poort in previous publications) and RB-YA (attributed to post-Howiesons Poort, Sibudan and Sibudu technocomplex in previous publications). Our principal aim was, first, to describe accurately the lithic technological changes, secondly, to correlate this description with other types of material culture and remains such as micromorphology, fauna and plant remains, and, finally, to propose hypotheses for the changes observed. The hypothesis of reduced mobility at Sibudu after 62 ka ago seems to fit with previous archaeological interpretations of situations with a similar type of pattern. Our purpose in this study was not to define culturally the RB to YA industries, attributed recently to the ‘Sibudan technocomplex’ [19], but to try to understand the mechanisms that were driving the technological changes described here through the cladistics analysis and the technological analysis.

With the evidence at hand for the period between 62 and 58 ka (see for a recent review [16]), there may be several independent technocomplexes arising in southern Africa. Given this situation, it is difficult to decide whether the RB to YA layers represent an independent technocomplex or whether they are related to technocomplexes elsewhere. For all the reasons explained in this technological study we prefer, at present, to retain the term post-Howiesons Poort for the assemblage in layers RB to YA at Sibudu. This study has also tried to make full use of data from the fine resolution multi-layered sediments of Sibudu Cave, which constitute a privileged window into the Middle Stone Age cultural behaviour of southern Africa.

## Supporting information

S1 File Description of the layers. (DOCX)

S2 File Autapomorphies and synapomorphies of the cladistics analysis. (DOCX)

S1 Table List of attributes for cladistics. (XLSX)

S2 Table All completed flakes. (XLSX)

S3 Table Normality tests for bipolar cores, flakes and blades and U-MannWhitney tests for different attributes. (XLSX)
