# Supplementary material for: Technological variability at Sibudu Cave: The end of Howiesons Poort and reduced mobility strategies after 62,000 years ago
Source: PLoS One. 2017 Oct 5;12(10):e0185845. doi: 10.1371/journal.pone.0185845 (PMC5628897; doi:10.1371/journal.pone.0185845)
Supplement: S1 File — (DOCX) [file pone.0185845.s001.docx]

**Grey Rocky (GR)**

GR is a fine silt and ash that lies loosely among small roof spalls. The sediment is relatively homogeneous compared with younger, brightly coloured lenses. Trampling in the past may have caused the homogeneous appearance of the sediments. Combustion features often comprise only white ash, others have white ash tops and grey or black bases, while a few have the three layer structure of rubified earth, black charcoal-rich ash and white ash. Most of the hearths are small and ephemeral, but one deep, wide hearth spans square B5 and reaches into the adjacent squares, while another large hearth occurs in B4/B5. No obvious bedding patches are present, but the presence of Cyperaceae seeds suggests that bedding was once present.

**Reddish Brown (RB)**

RB does not cover all squares, but has one clear combustion feature.

**Brown under Yellow Ash 2(i)**

The layer is characterised by millimetre thin lenses of white, black and reddish-yellow ash from an accumulation of combustion events. Seven combustion features occur in the area excavated and they include hearths and burned bedding areas (Wadley et al. 2011). Organic petrological analysis produced reflectance measurements implying that herbaceous material was burned at maximum temperatures of between 289 and 319°C (Bentsen 2014). This supports the interpretation of grass and sedge bedding burnt for site maintenance purposes.

**Brown under Yellow Ash 2**

BYA2 was recognised in squares B5, B6, C5 and C6, directly below Layer YA2. YA2 was thinner here than in squares B4 and C4, where YA2 was divided from layer YA2(i) by the lens called GMOD under YA2.

**Brown under Yellow Ash**

There are many calcined bone fragments and, in addition, abundant charred seeds and large pieces of charcoal. C6 has lots of gypsum crystals, and few seed fragments and small fragments of charcoal were recovered here.

**Mottled Grey under YA2**

Small bone fragments are calcined or lightly burnt and there are only tiny fragments of charcoal. Nonetheless, many charred seeds were collected.

**Yellow Ash 2(i), Yellow Ash 2 and YA**

These yellow, loose, uncemented ashy silts (YA2i, YA2, YA) are similar in composition: wood ash is an important constituent and there are many phytoliths from woody plants (yet few grass phytoliths are included) (Schiegl et al. 2004). Apatite and tri-calcium-phosphate, a mineral found in incinerated bone, is common (Schiegl and Conard 2006). Relatively little gypsum was found in these silty layers, but calcite is also not abundant. Importantly, the Yellow Ash layers seem to lack the burnt, laminar monocotyledon bedding layers associated with many of the other 58 ka occupations. In **YA2i** much highly calcined bone and charred seed fragments occur, but little charcoal. In **YA2** there are lots of calcined bone fragments, some large, but only small pieces of charcoal. YA2 is particularly rich in charred seeds, especially Type 5, which may be an *Olea* sp. (Sievers 2006). In **YA** where the hearth in C6 is cemented ash (10YR 8/6 yellow) with gypsum and little organic material. Rodent burrows cut horizontally across squares C5 and C6. There is a lot of highly burnt, smashed bone, small fragments of charcoal and many charred seed fragments.
