# Supplementary material for: Technological variability at Sibudu Cave: The end of Howiesons Poort and reduced mobility strategies after 62,000 years ago
Source: PLoS One. 2017 Oct 5;12(10):e0185845. doi: 10.1371/journal.pone.0185845 (PMC5628897; doi:10.1371/journal.pone.0185845)
Supplement: S2 File — (DOCX) [file pone.0185845.s002.docx]

**Tree 1**

GS :

No autapomorphies

GR :

Presence_of_centripetal_or_multifacial_cores_dolerite (17): Absence --> Presence

Presence_of_centripetal_or_multifacial_cores_quartz (19): Presence --> Absence

Presence_of_bladelet_cores_quartz (22): Absence --> Presence

Presence_of_Levallois_flakes_dolerite_or_hornfels (33): Absence --> Presence

RB :

Second_most_important__rock_type_Hornfels (6): Absence --> Presence

Presence_of_core_on_flake_hornfels (25): Absence --> Presence

BYA2i :

Presence_of_bladelet_cores_quartz (22): Absence --> Presence

Dominance_of_bifacial_pieces (61): Absence --> Presence

YA2I :

Main_rock_type_Dolerite (0): Presence --> Absence

Main_rock_type_Quartz (2): Absence --> Presence

Second_most_important__rock_type_Dolerite (5): Absence --> Presence

Main_retouched_rock_type__Quartzite (13): Absence --> Presence

Presence_of_prismatic_blades_hornfels (32): Absence --> Presence

Presence_of_discoidal_flakes__(pseudolevallois_flakes,_edge_of_the_core)_quartz (36): Absence --> Presence

Presence_of_backed_pieces (55): Presence --> Absence

BYA2 :

Main_rock_type_Sandstone (4): Absence --> Presence

Main_retouched_rock_type__Hornfels (11): Absence --> Presence

Presence_of_core_on_flake_dolerite (26): Absence --> Presence

Presence_of_prismatic_blades_hornfels (31): Presence --> Absence

Presence_of_bladelets_quartz (41): Absence --> Presence

Presence_of_backed_pieces (55): Presence --> Absence

Mottled :

Main_retouched_rock_type__Quartzite (13): Absence --> Presence

Presence_of_bladelets_quartzite (40): Absence --> Presence

Dolerite:_dominance_of_parallel_shapes (54): Presence --> Absence

Presence_of_grindstones_rubber_stones (63): Absence --> Presence

YA2 :

Presence_of_centripetal_or_multifacial_cores_hornfels (18): Absence --> Presence

Preference_on_blades_for_retouched_blanks (56): Absence --> Presence

Preference_on_flakes_for_retouched_blanks (57): Presence --> Absence

BYA :

Presence_of_prismatic_blades_hornfels (32): Absence --> Presence

Presence_of_false_semicrest_(change_in_the_direction_of_knapping) (43): Presence --> Absence

Presence_of_backed_pieces (55): Presence --> Absence

Presence_of_grindstones_rubber_stones (63): Absence --> Presence

YA :

Presence_of_knapping_in_crystal_quartz (15): Absence --> Presence

Presence_of_bipolar_cores_quartzite (28): Presence --> Absence

Presence_of_discoidal_flakes__(pseudolevallois_flakes,_edge_of_the_core)_dolerite (34): Absence --> Presence

Presence_of_discoidal_flakes__(pseudolevallois_flakes,_edge_of_the_core)_hornfels (35): Absence --> Presence

Presence_of_discoidal_flakes__(pseudolevallois_flakes,_edge_of_the_core)_quartz (36): Absence --> Presence

Presence_of_discoidal_flakes__(pseudolevallois_flakes,_edge_of_the_core)_quartzite (37): Absence --> Presence

Presence_of_false_semicrest_(change_in_the_direction_of_knapping) (43): Presence --> Absence

Dolerite:_dominance_of_unidirectional_scar_pattern_for_flakes (51): Presence --> Absence

Dolerite:_dominance_of_centripetal_subcentripetal_scar_pattern_for_flakes (52): Absence --> Presence

Dolerite:_dominance_of_parallel_shapes (54): Presence --> Absence

Node 11 :

No synapomorphies

Node 12 :

Main_retouched_rock_type_Dolerite (10): Absence_ --> Presence

Presence_of_big_notches (59): Presence --> Absence

Node 13 :

Main_retouched_rock_type__Quartz (12): Presence --> Absence

Presence_of_bladelets_quartz (41): Presence --> Absence

Presence_of_bifacial_pieces (58): Presence --> Absence

Dominance_of_other_types (62): Absence --> Presence

Node 14 :

Dolerite:_dominance_of_parallel_shapes (54): Absence --> Presence

Node 15 :

Presence_of_knapping_in_crystal_quartz (15): Presence --> Absence

Presence_of_centripetal_or_multifacial_cores_hornfels (18): Presence --> Absence

Presence_of_bladelet_cores_hornfels (20): Presence --> Absence

Presence_of_prismatic_blade_cores_hornfels (23): Presence --> Absence

Presence_of_prismatic_blade_cores_dolerite (24): Presence --> Absence

Presence_of_core_on_flake_hornfels (25): Presence --> Absence

Presence_of_core_on_flake_dolerite (26): Presence --> Absence

Presence_of_bipolar_cores_quartz (27): Presence --> Absence

Presence_of_HP_cores_hornfels (29): Presence --> Absence

Presence_of_bladelets_dolerite (38): Presence --> Absence

Presence_of_crests_or_semicrests (42): Presence --> Absence

Presence_of_autonomous_flake_production_in_quartzite (47): Absence --> Presence

Presence_of_recycling_from_freehand_quartz_cores_to_bipolar_cores (50): Presence --> Absence

Preference_on_blades_for_retouched_blanks (56): Presence --> Absence

Preference_on_flakes_for_retouched_blanks (57): Absence --> Presence

Dominance_of_backed_pieces (60): Presence --> Absence

Node 16 :

Second_most_important__rock_type__Quartz (7): Absence --> Presence

Dolerite:_dominance_of_unidirectional_scar_pattern_for_flakes (51): Presence --> Absence

Dolerite:_dominance_of_centripetal_subcentripetal_scar_pattern_for_flakes (52): Absence --> Presence

Presence_of_big_notches (59): Presence --> Absence

Node 17 :

Main_rock_type_Dolerite (0): Presence --> Absence

Main_rock_type_Quartzite (3): Absence --> Presence

Second_most_important__rock_type_Dolerite (5): Absence --> Presence

Presence_of_bladelets_hornfels (39): Presence --> Absence

Presence_of_false_semicrest_(change_in_the_direction_of_knapping) (43): Presence --> Absence

Node 18 :

Second_most_important__rock_type__Quartz (7): Absence --> Presence

Main_retouched_rock_type__Hornfels (11): Absence --> Presence

Presence_of_centripetal_or_multifacial_cores_dolerite (17): Absence --> Presence

Presence_of_bladelets_dolerite (38): Absence --> Presence

Presence_of_grindstones_rubber_stones (63): Absence --> Presence
